# Supplementary material for: as-Indaceno[3,2,1,8,7,6-ghijklm]terrylene as a near-infrared absorbing C70-fragment
Source: Nat Commun. 2020 Aug 3;11:3873. doi: 10.1038/s41467-020-17684-6 (PMC7400669; doi:10.1038/s41467-020-17684-6)
Supplement: Supplementary file 1 — Supplementary Information [file 41467_2020_17684_MOESM1_ESM.pdf]

Supplementary Information

as-Indaceno[3,2,1,8,7,6-*ghijklm*]terrylene as a near-infrared absorbing C_{70}-fragment

Tanaka *et al.*

Supplementary Figures

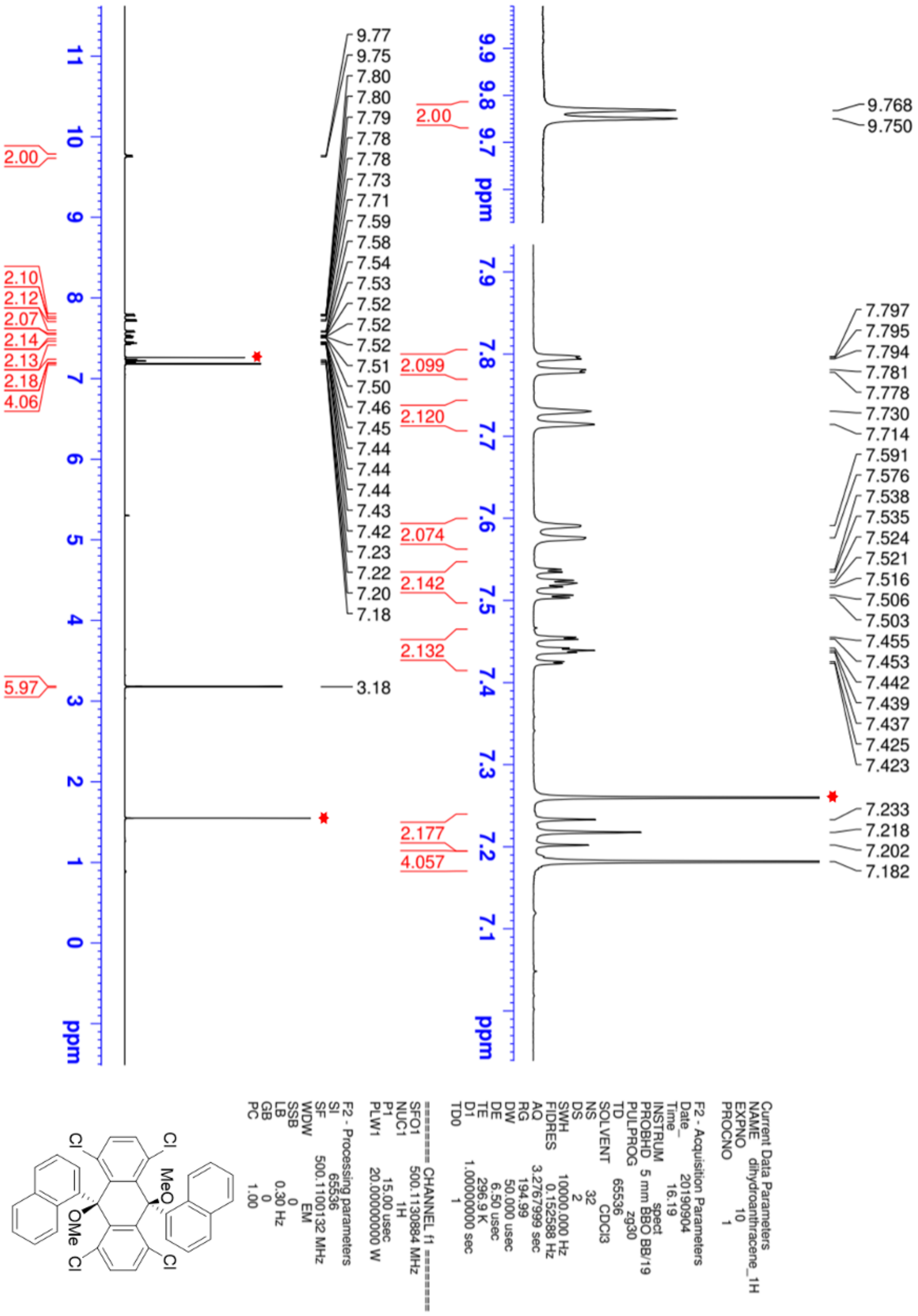

Supplementary Figure 1. ^{1}H NMR spectrum (500 MHz) of **5** in $CDCl_3$.

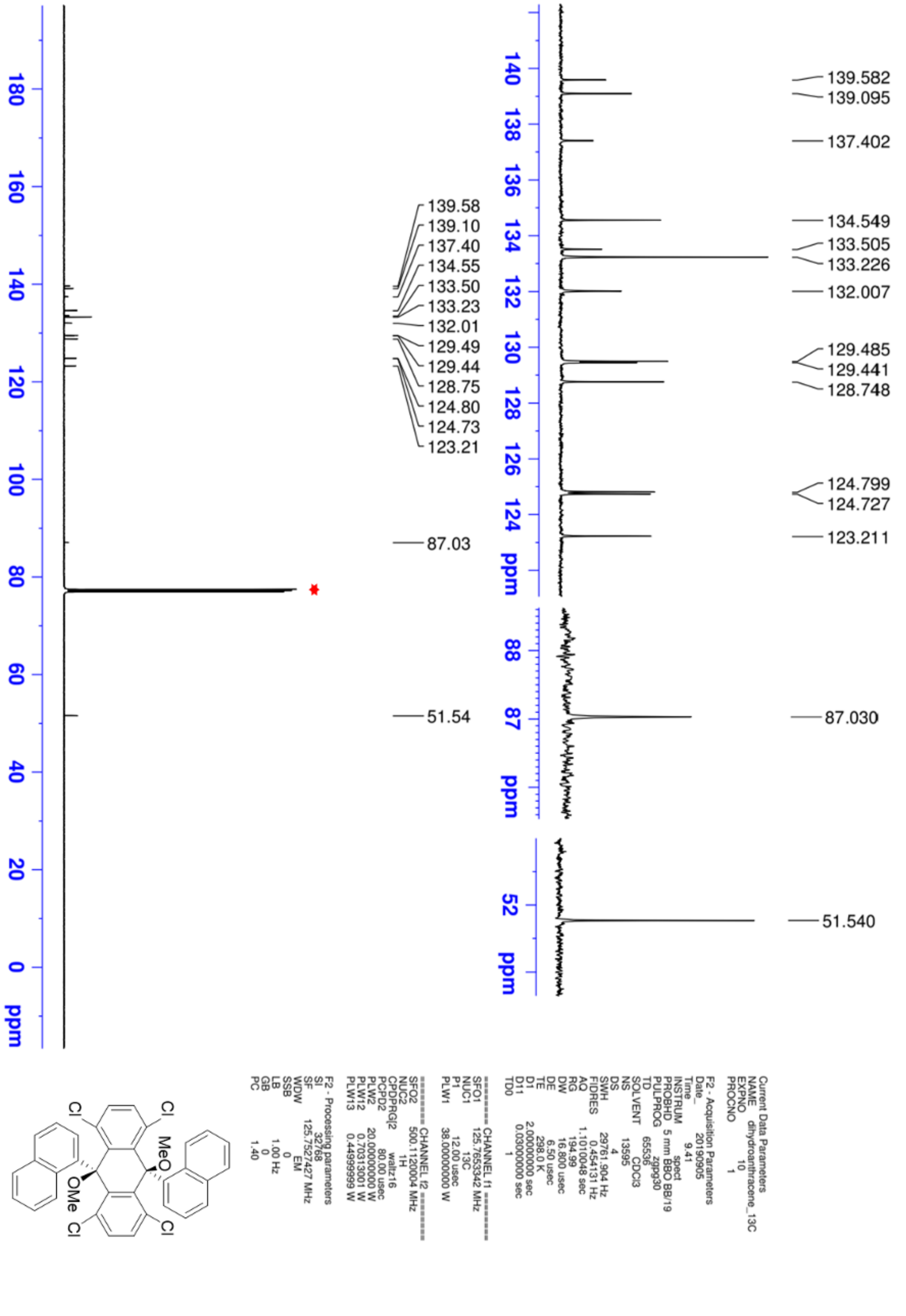

Supplementary Figure 2. ^{13}C NMR spectrum (126 MHz) of **5** in $CDCl_3$.

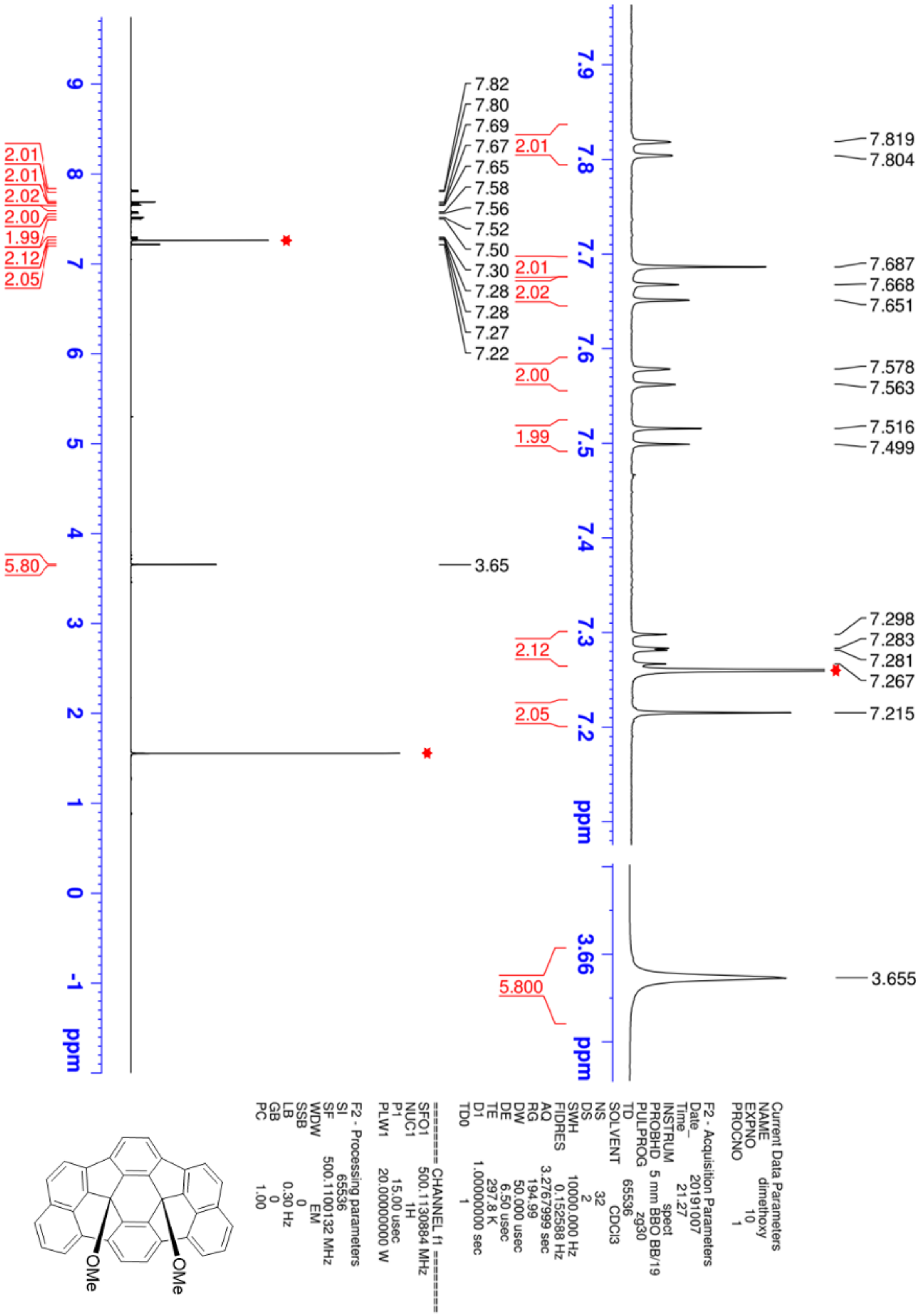

Supplementary Figure 3. ^{1}H NMR spectrum (500 MHz) of **7** in $CDCl_3$.

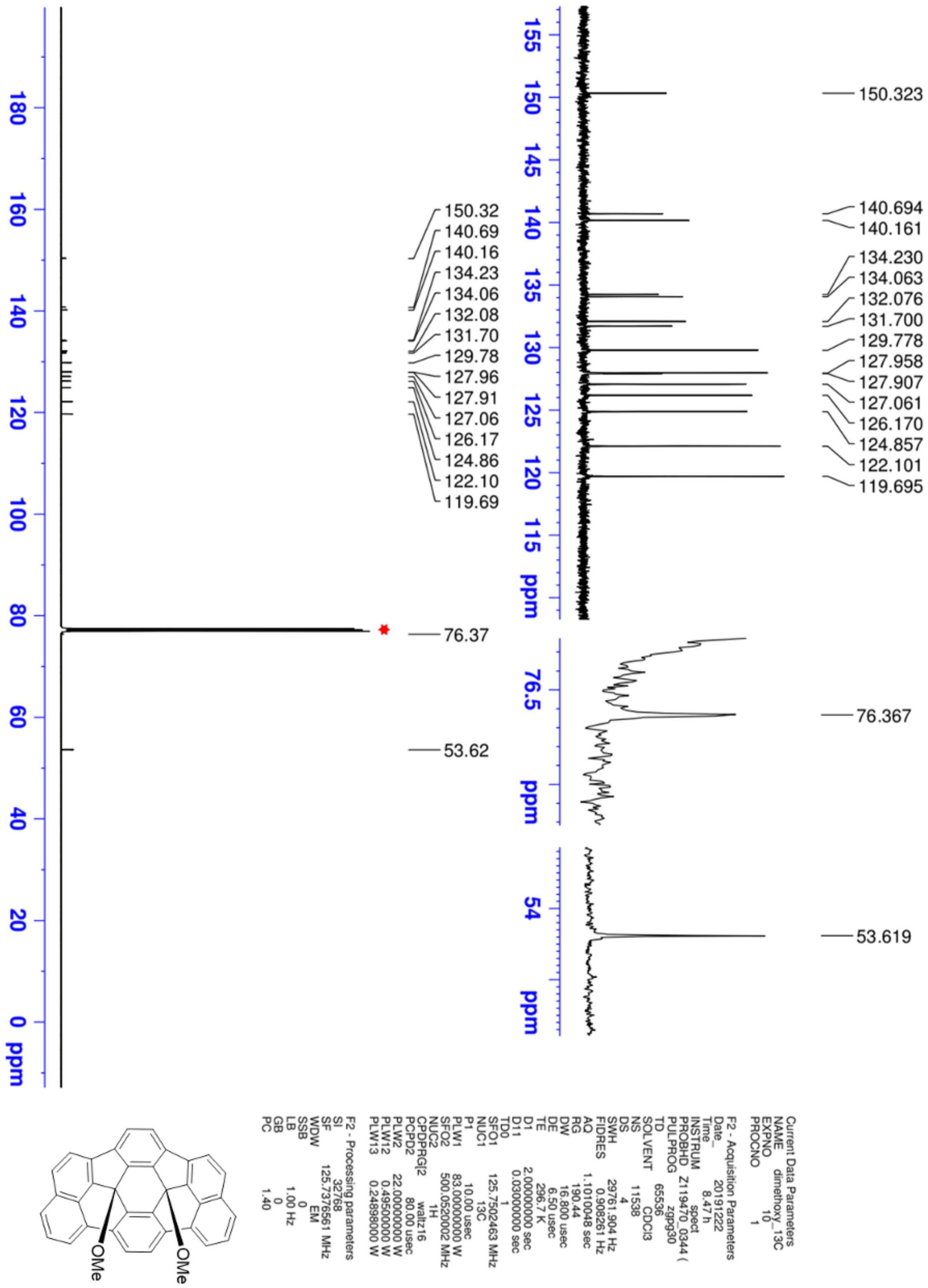

Supplementary Figure 4. ^{13}C NMR spectrum (126 MHz) of **7** in $CDCl_3$.

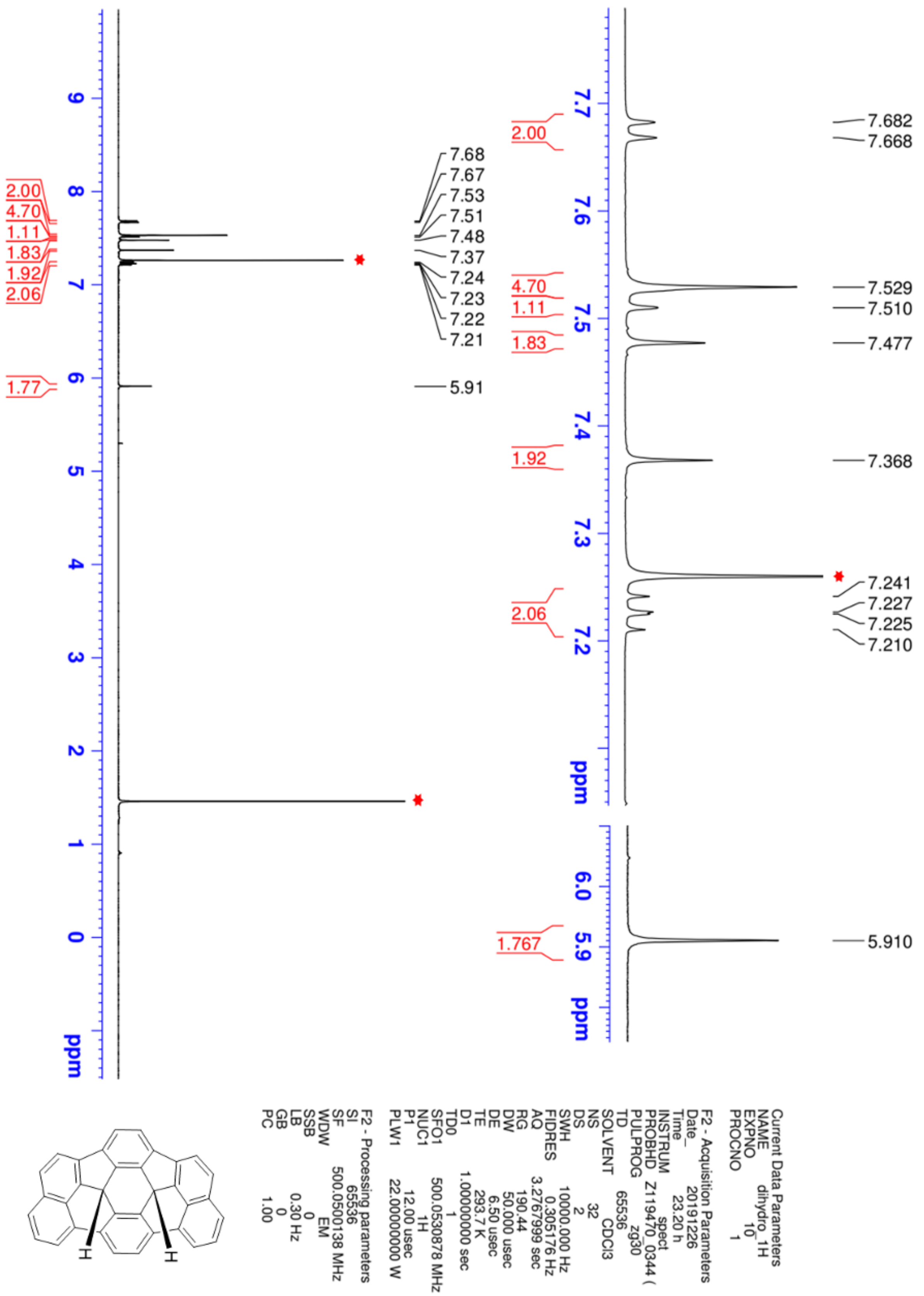

Supplementary Figure 5. ^{1}H NMR spectrum (500 MHz) of **8** in $CDCl_3/CS_2$ (1/1).

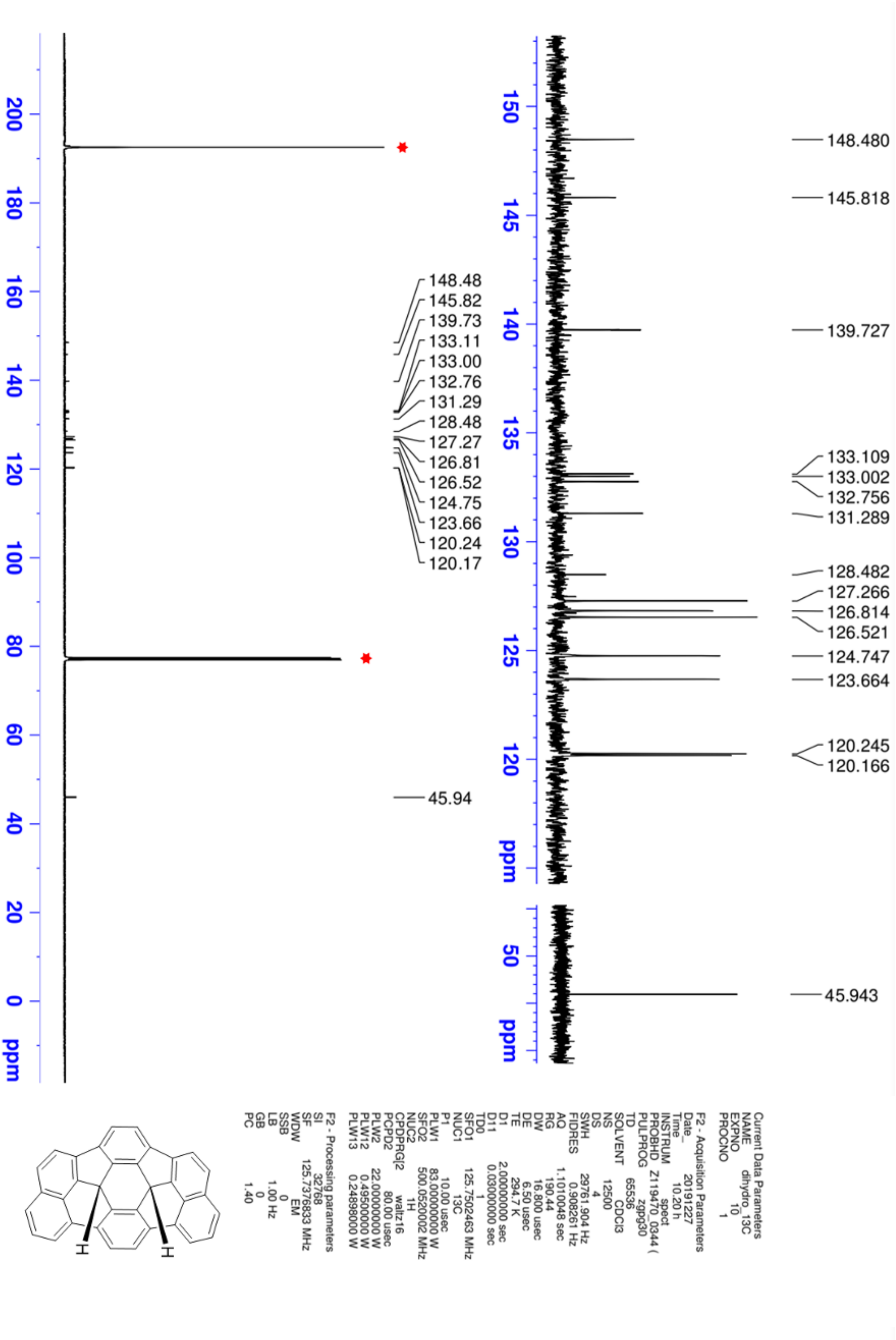

Supplementary Figure 6. ^{13}C NMR spectrum (126 MHz) of **8** in $CDCl_3/CS_2$ (1/1).

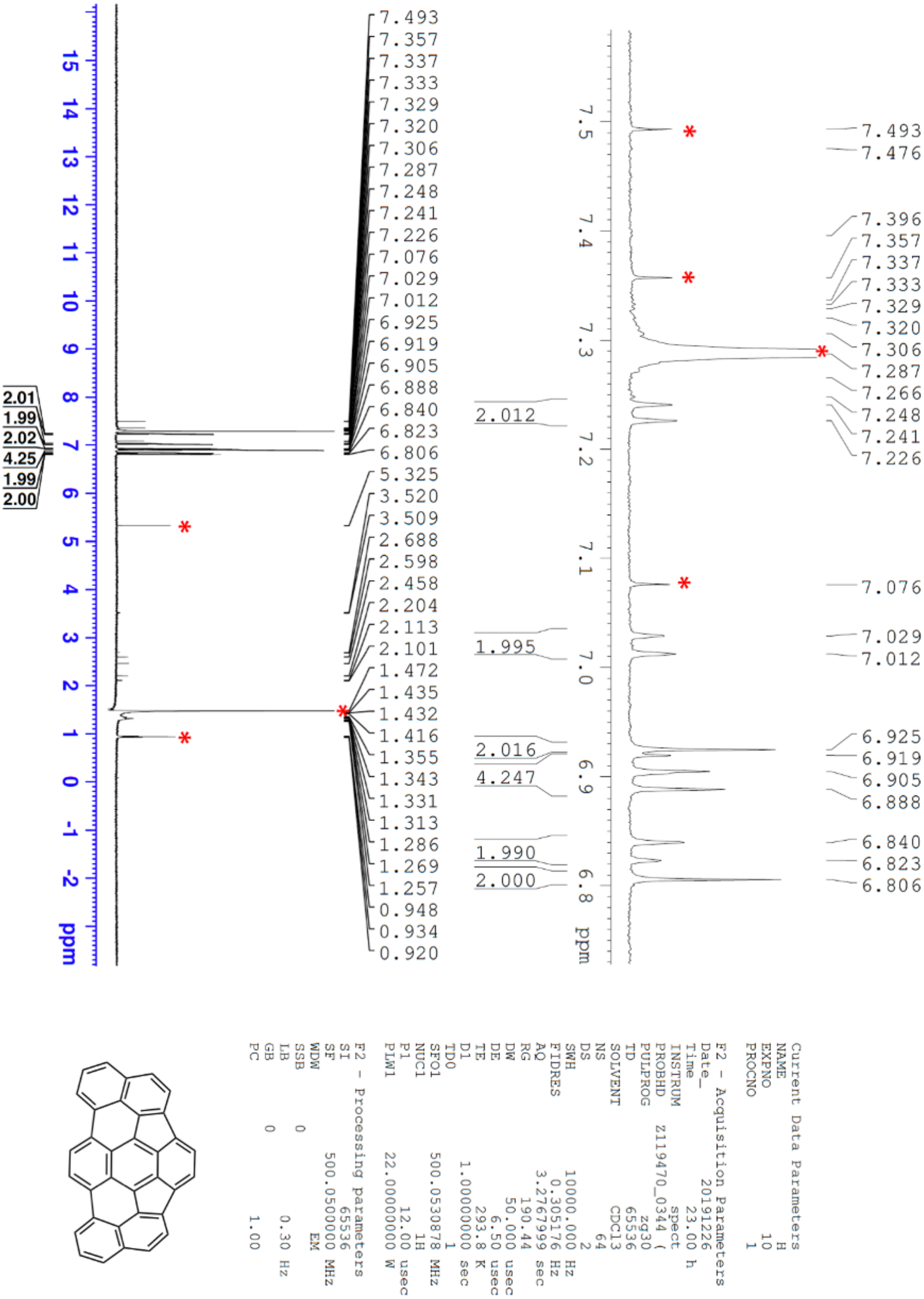

Supplementary Figure 7. ^{1}H NMR spectrum (500 MHz) of **3** in $CDCl_3/CS_2$ (1/1).

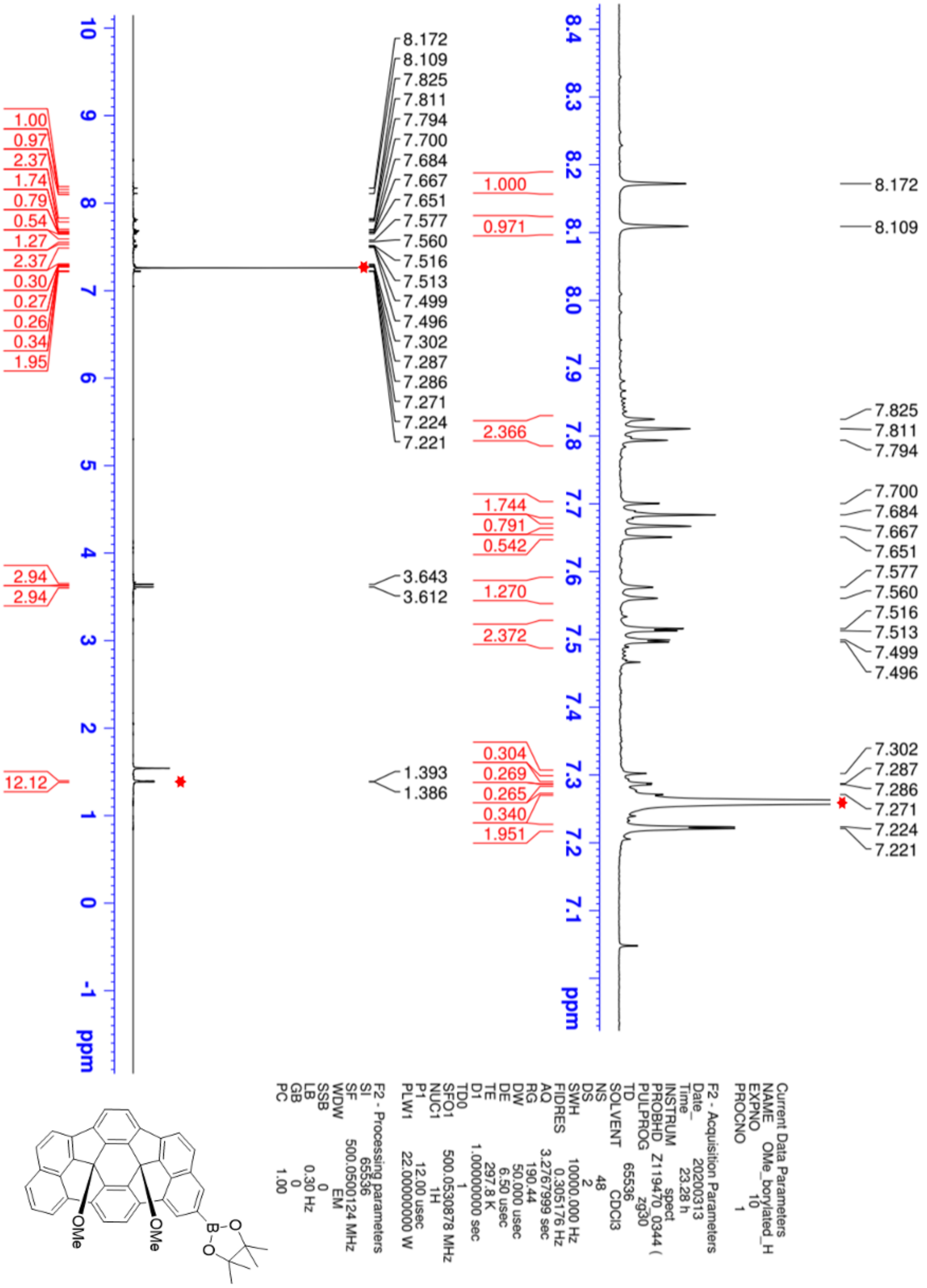

Supplementary Figure 8. ^{1}H NMR spectrum (500 MHz) of **10** in $CDCl_3$.

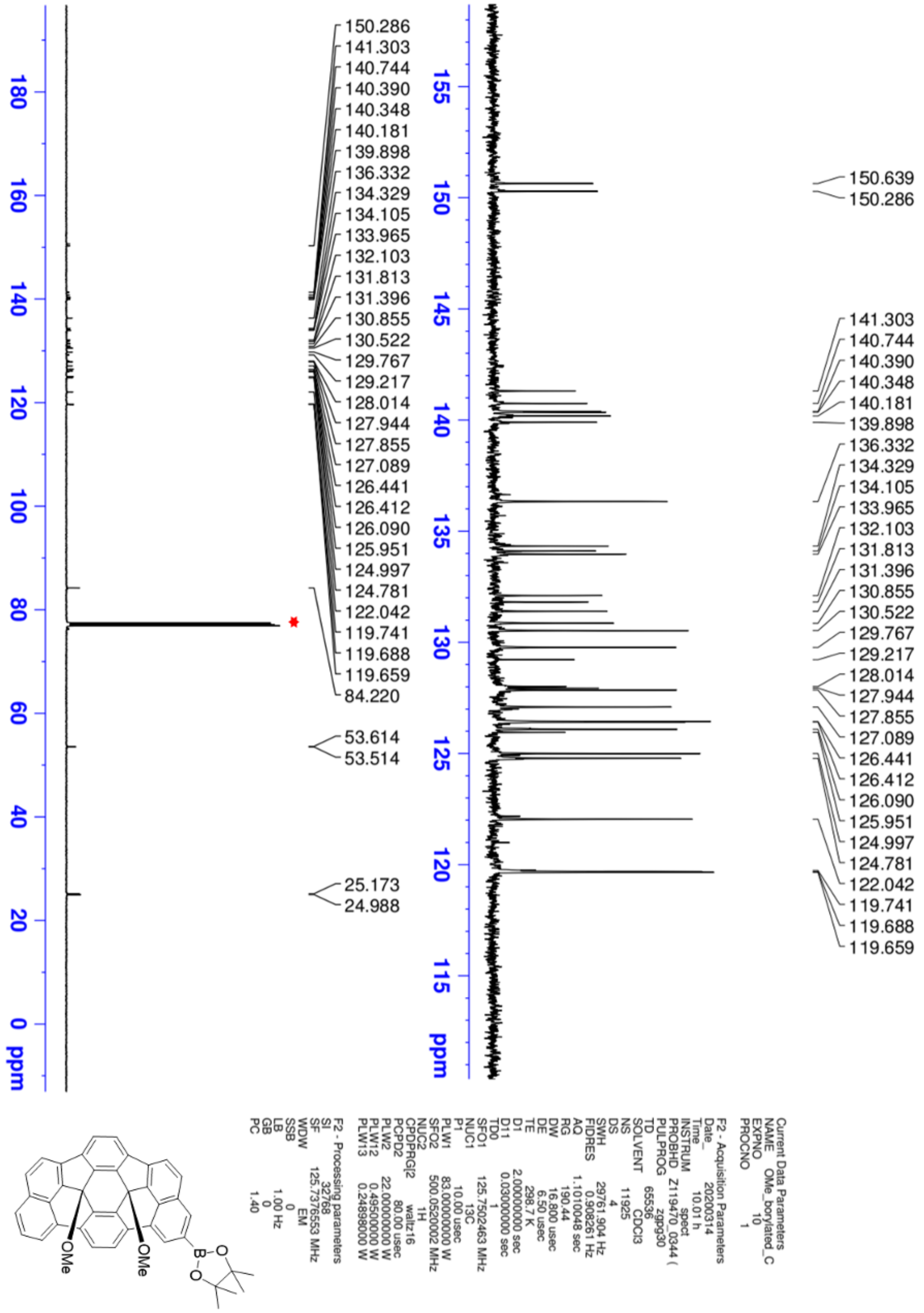

Supplementary Figure 9. ^{13}C NMR spectrum (126 MHz) of **10** in $CDCl_3$.

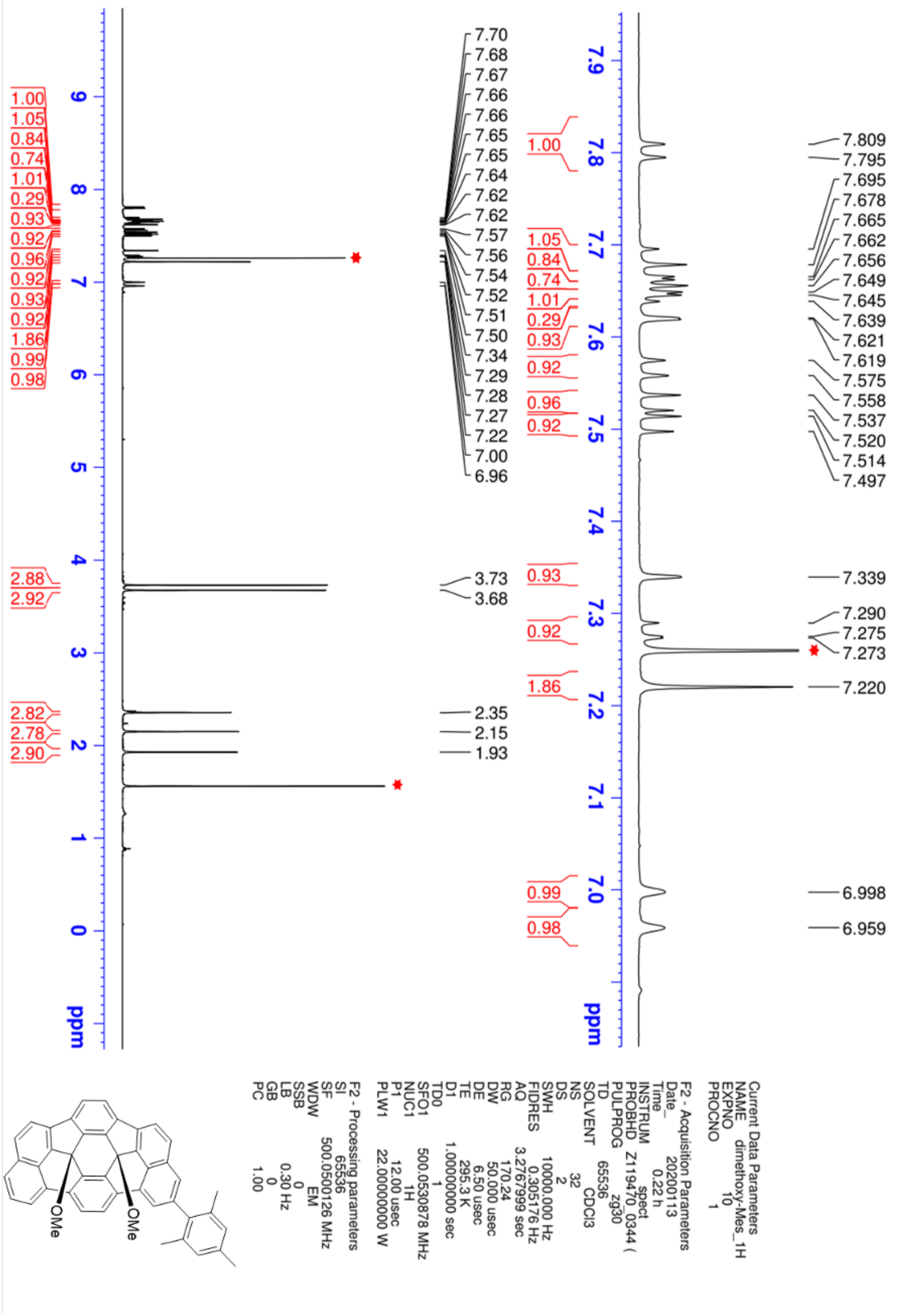

Supplementary Figure 10. ^{1}H NMR spectrum (500 MHz) of **11** in $CDCl_3$.

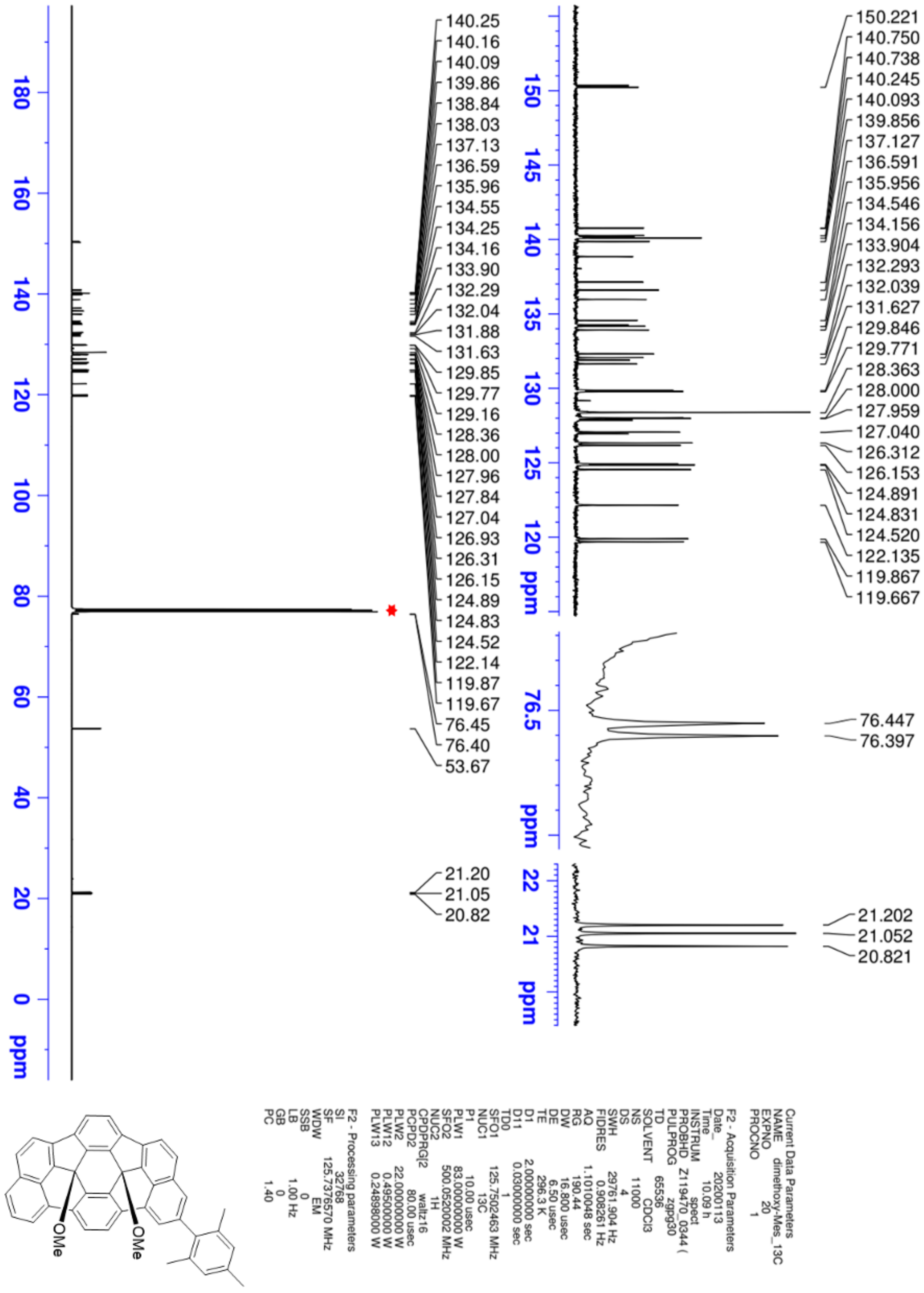

Supplementary Figure 11. ^{13}C NMR spectrum (126 MHz) of **11** in $CDCl_3$.

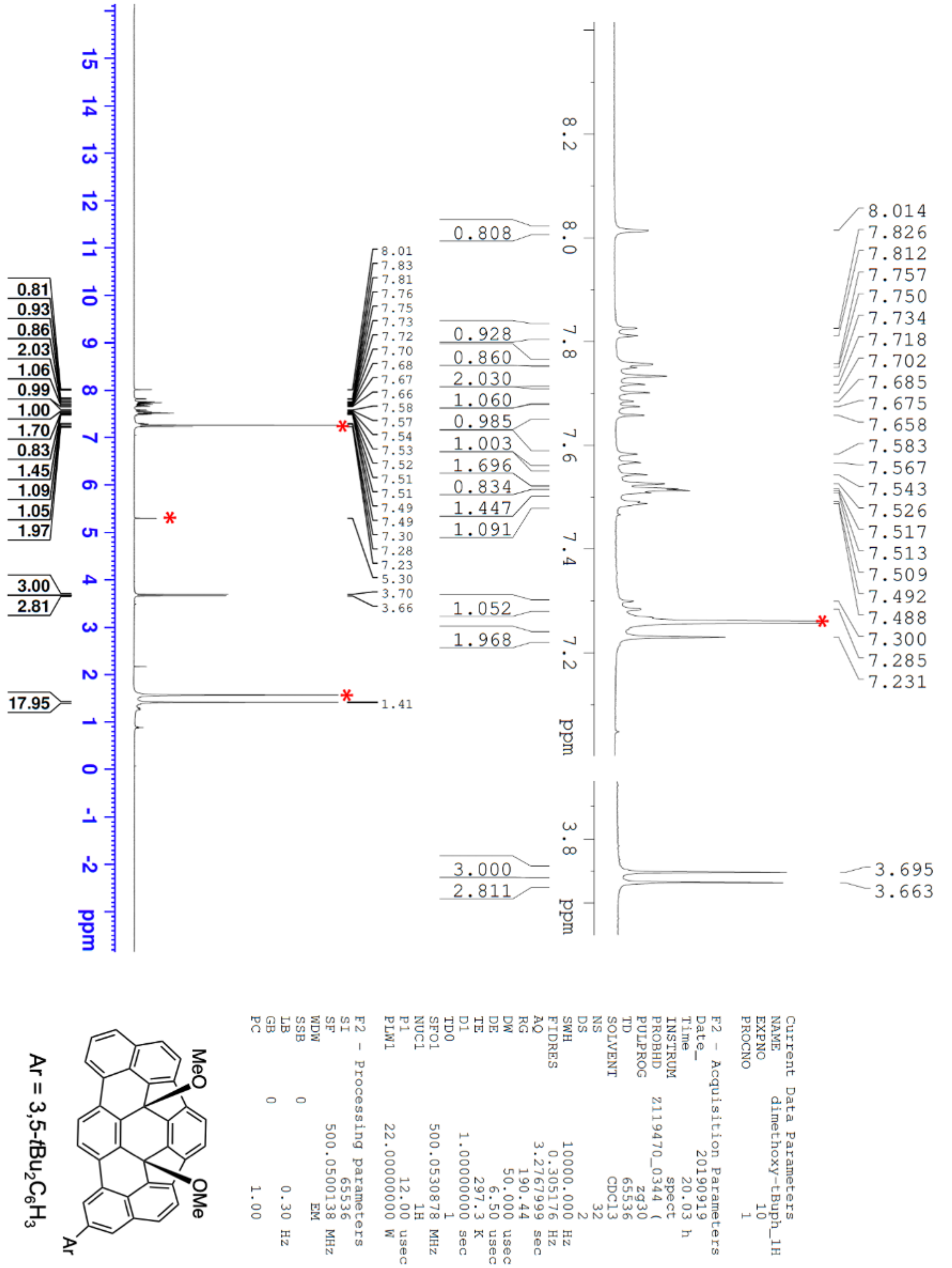

Supplementary Figure 12. ^{1}H NMR spectrum (500 MHz) of **12** in $CDCl_3$.

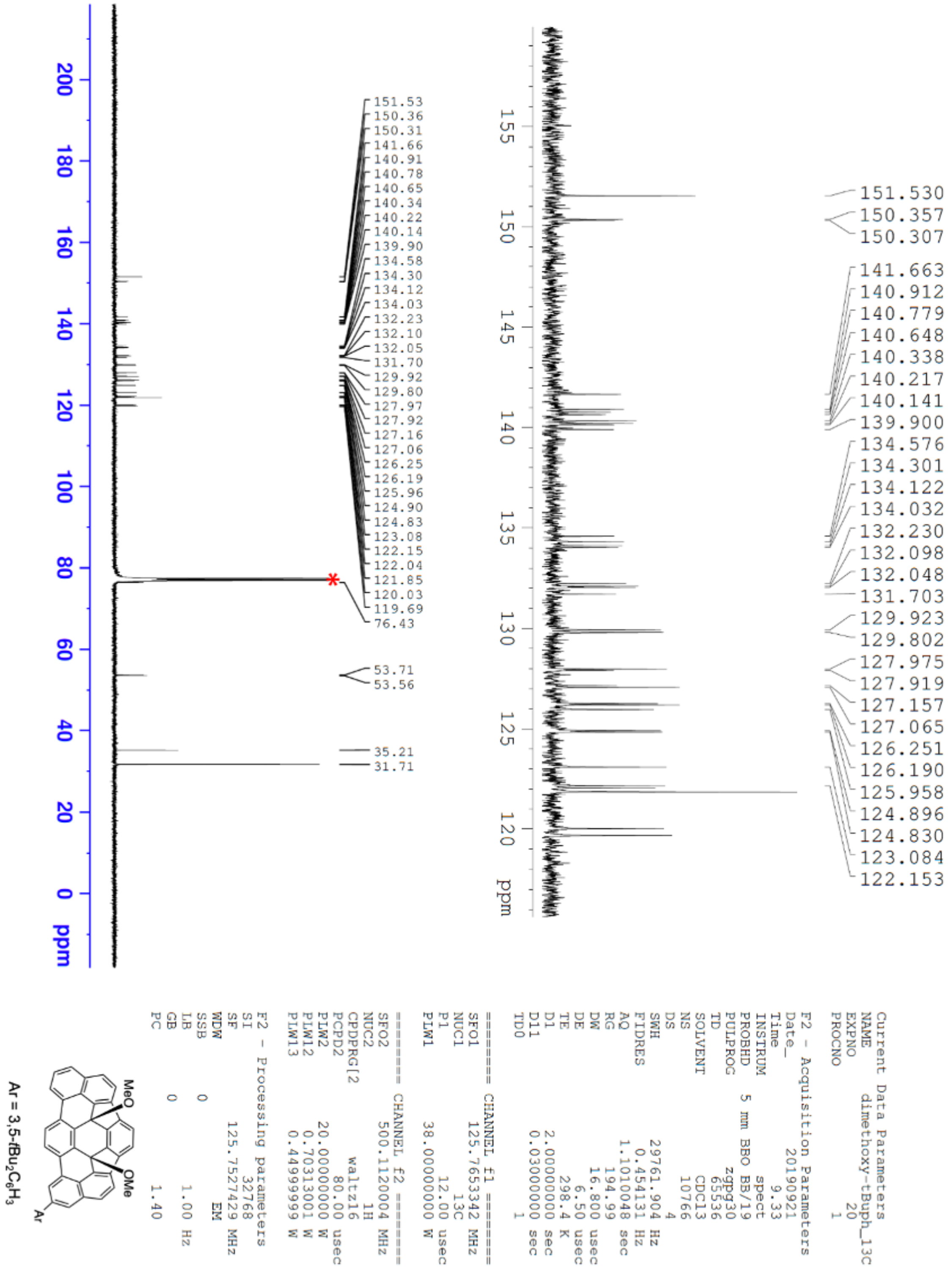

Supplementary Figure 13. ^{13}C NMR spectrum (126 MHz) of **12** in $CDCl_3$.

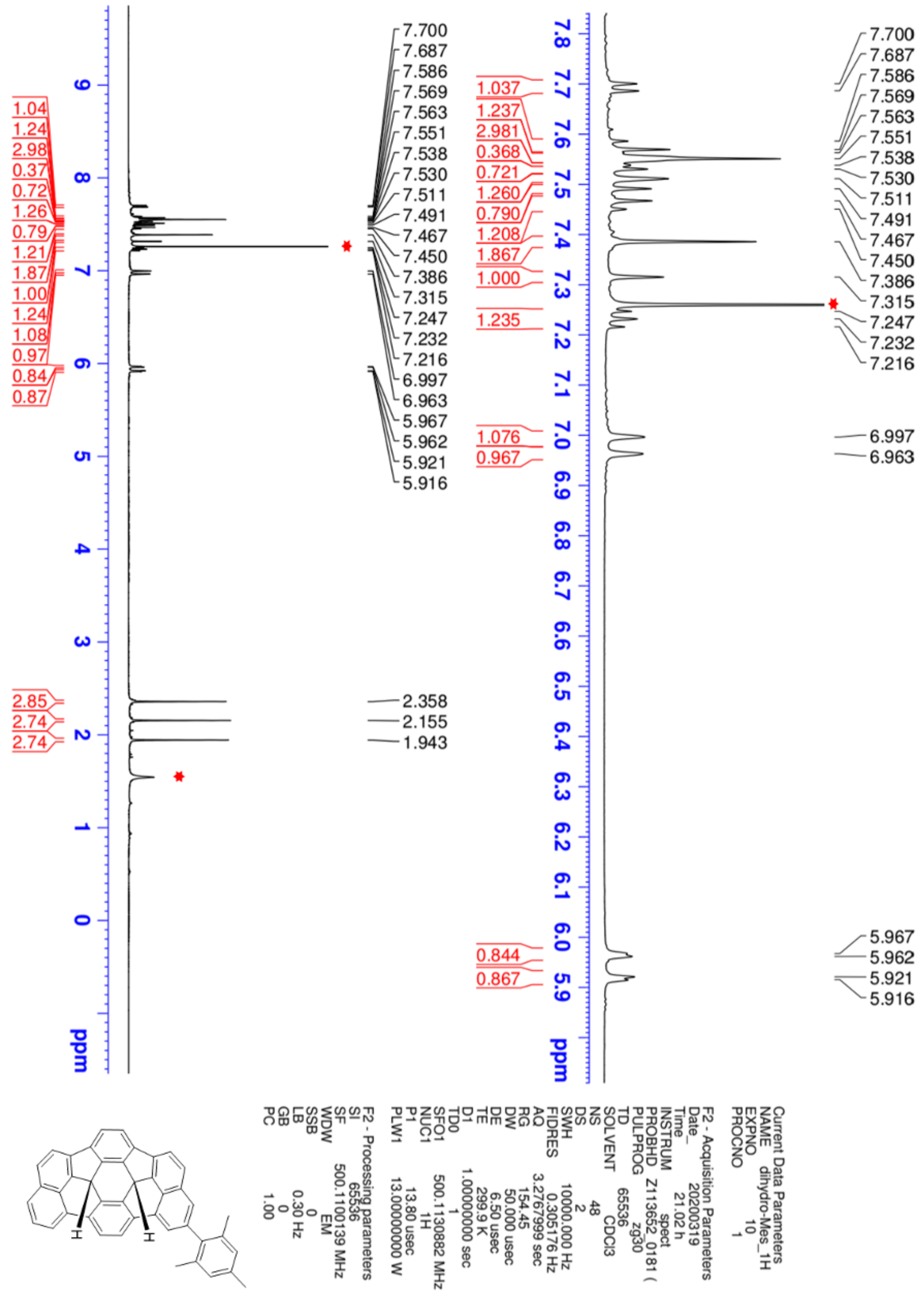

Supplementary Figure 14. ^{1}H NMR spectrum (500 MHz) of **13** in $CDCl_3$.

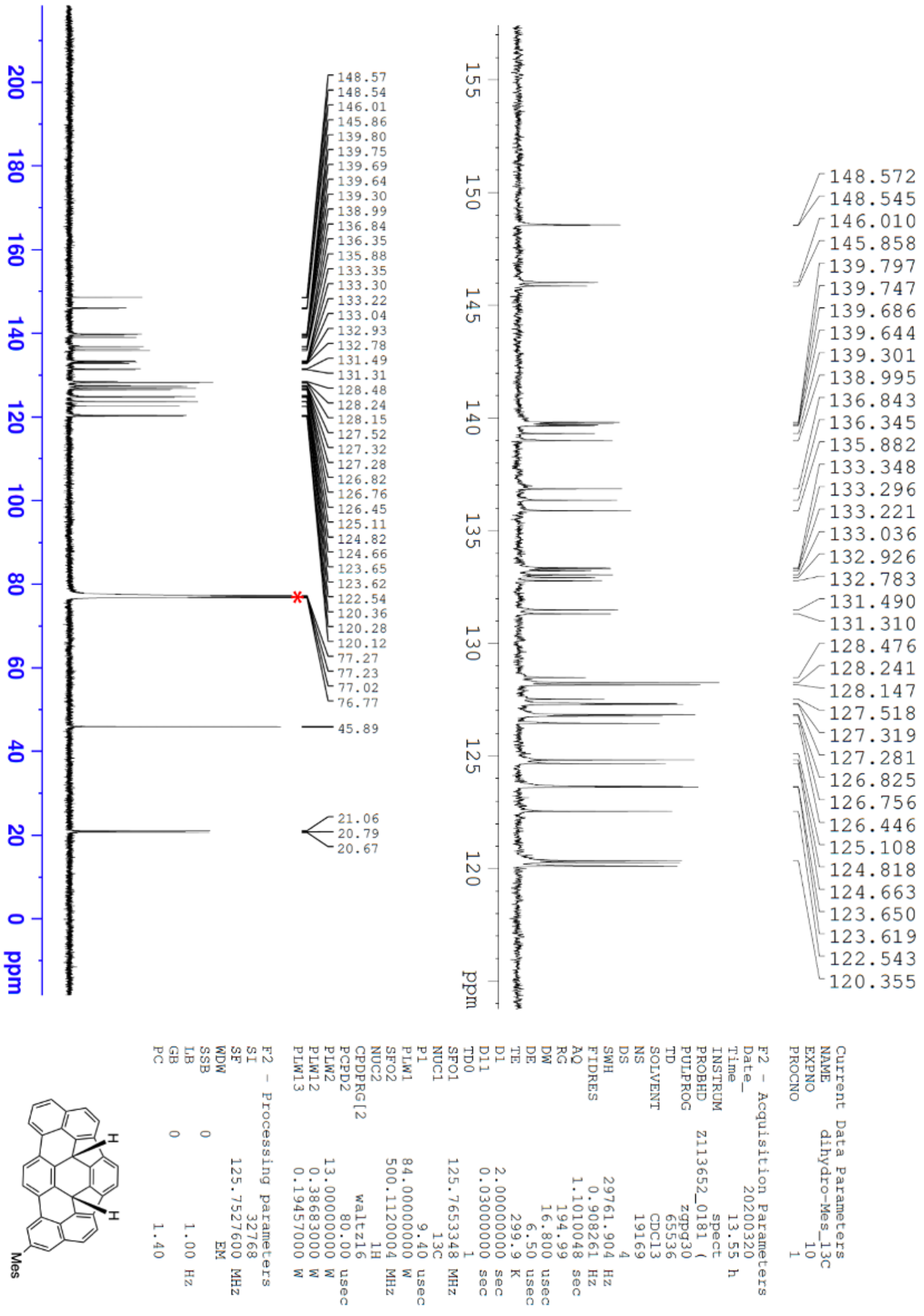

Supplementary Figure 15. ^{13}C NMR spectrum (126 MHz) of **13** in $CDCl_3$.

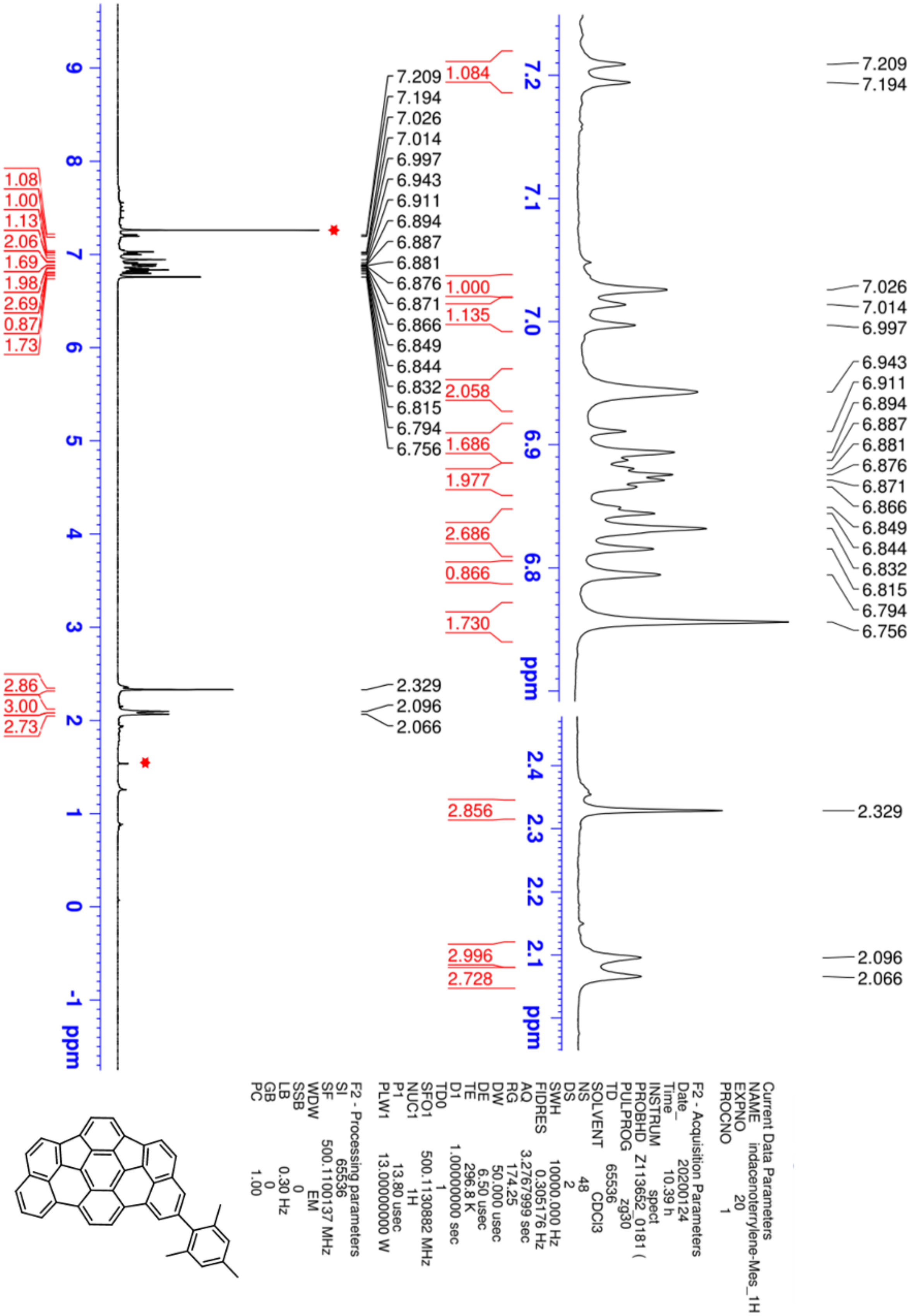

Supplementary Figure 16. ^{1}H NMR spectrum (500 MHz) of **9** in $CDCl_3$.

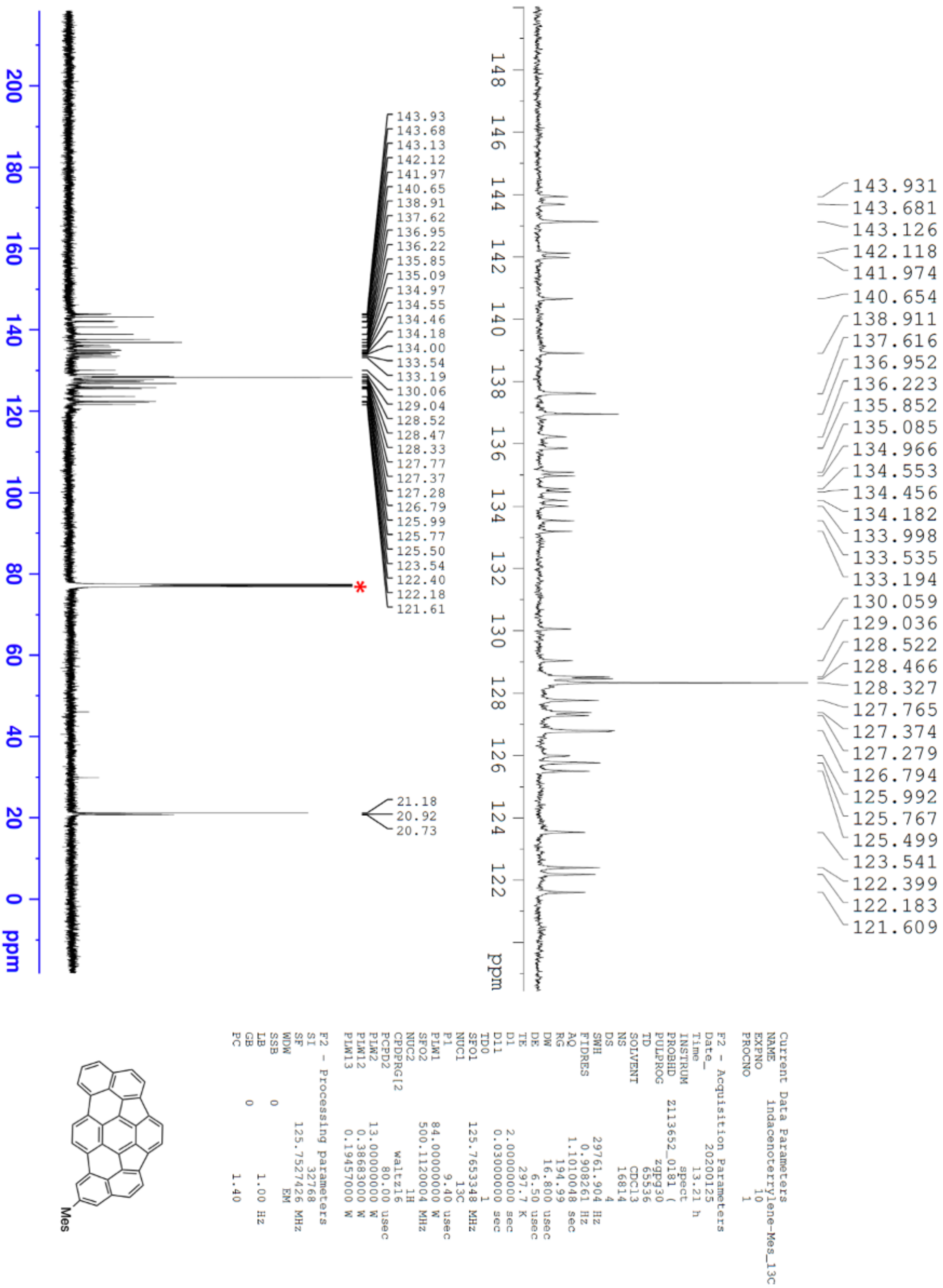

Supplementary Figure 17. ^{13}C NMR spectrum (126 MHz) of **9** in $CDCl_3$.

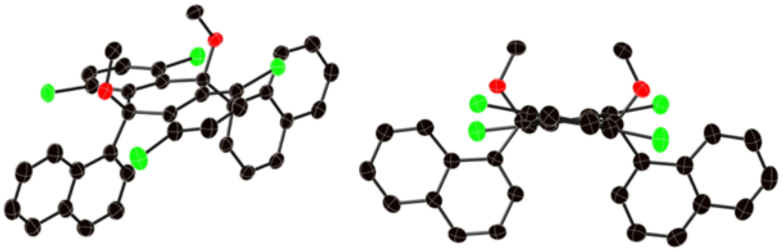

Supplementary Figure 18. X-ray crystal structure of **5**. Thermal ellipsoids are drawn at the 50% probability level.

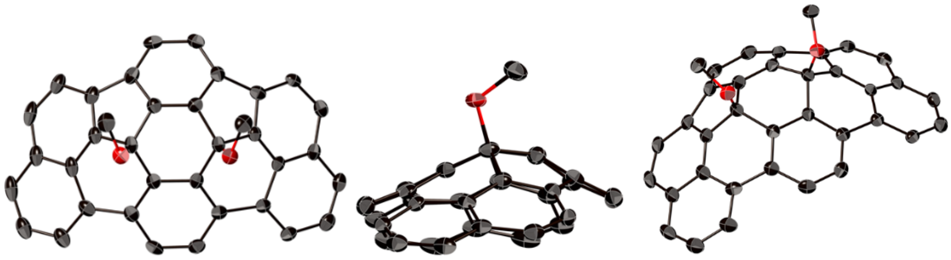

Supplementary Figure 19. X-ray crystal structure of **7**. Thermal ellipsoids are drawn at the 50% probability level.

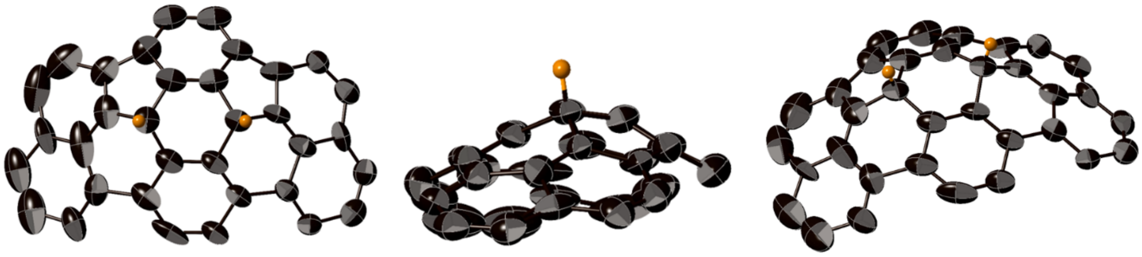

Supplementary Figure 20. X-ray crystal structure of **8**. Thermal ellipsoids are drawn at the 50% probability level.

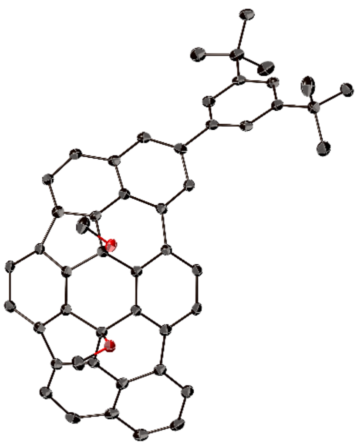

Supplementary Figure 21. X-ray crystal structure of **12**. Thermal ellipsoids are drawn at the 50% probability level.

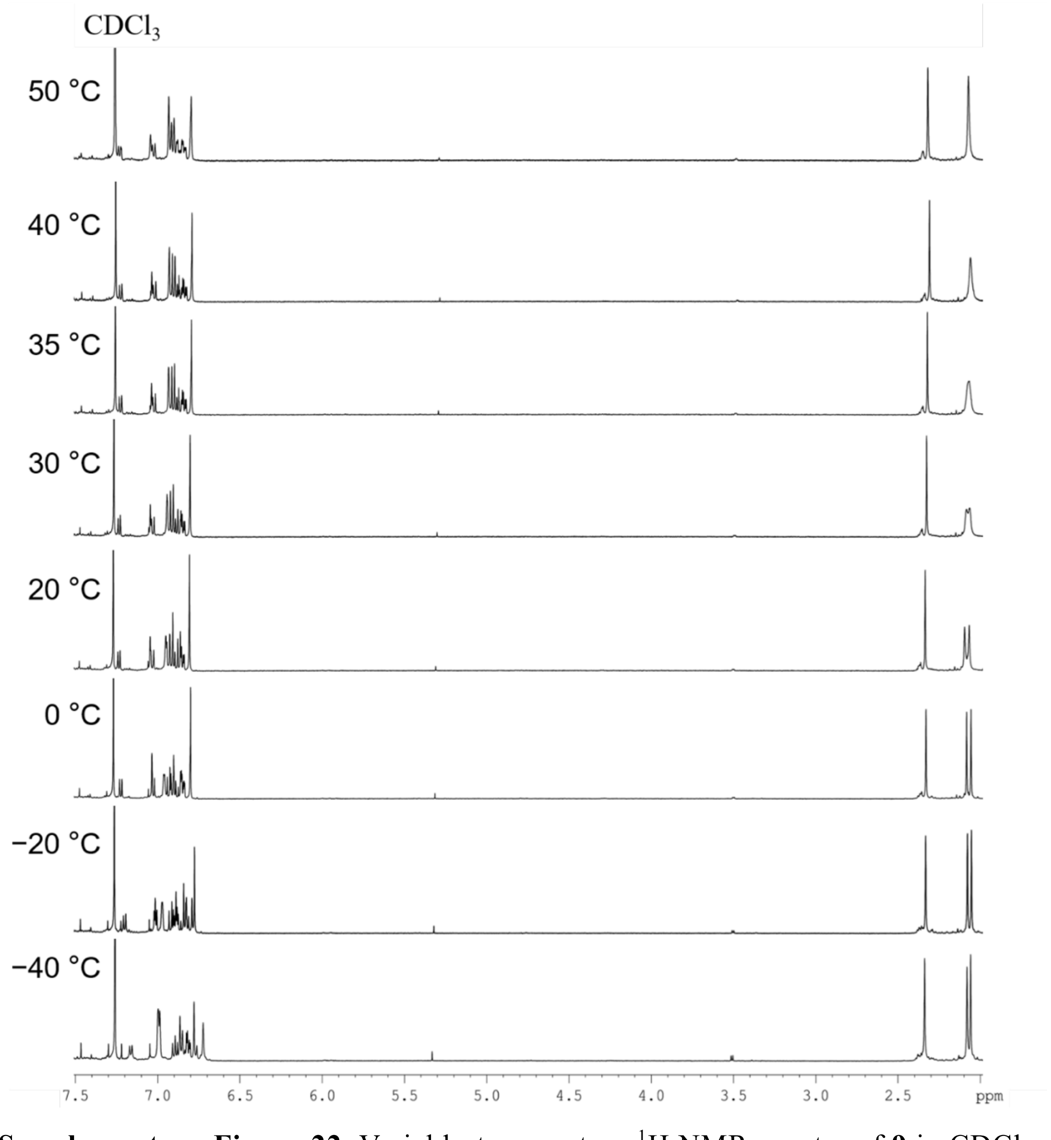

Supplementary Figure 22. Variable-temperature ^{1}H NMR spectra of **9** in $CDCl_3$.

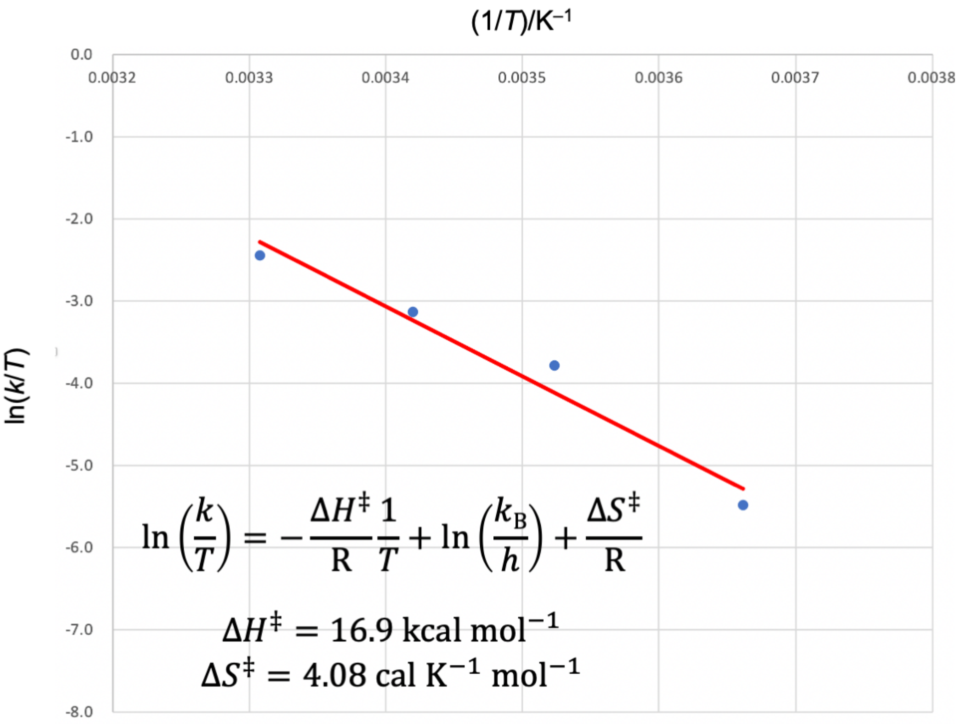

Supplementary Figure 23. Eyring plot for determination of the bowl-to-bowl inversion barrier

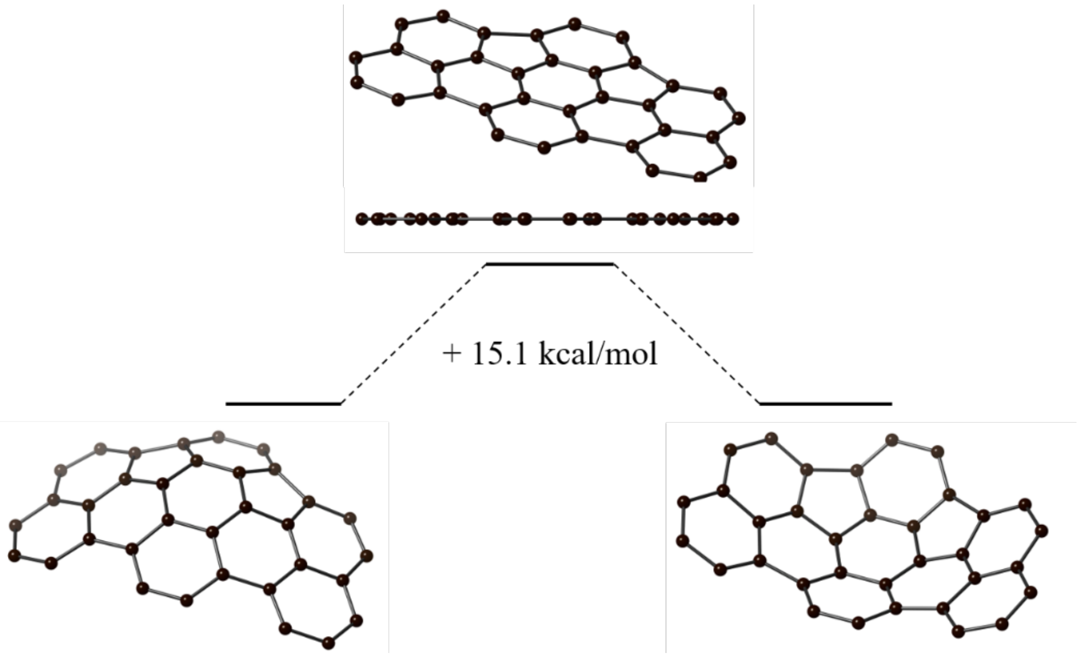

Supplementary Figure 24. Calculated inversion behavior of **3** (CAM-B3LYP/6-311+G(d,p)//B3LYP/6-31G(d)).

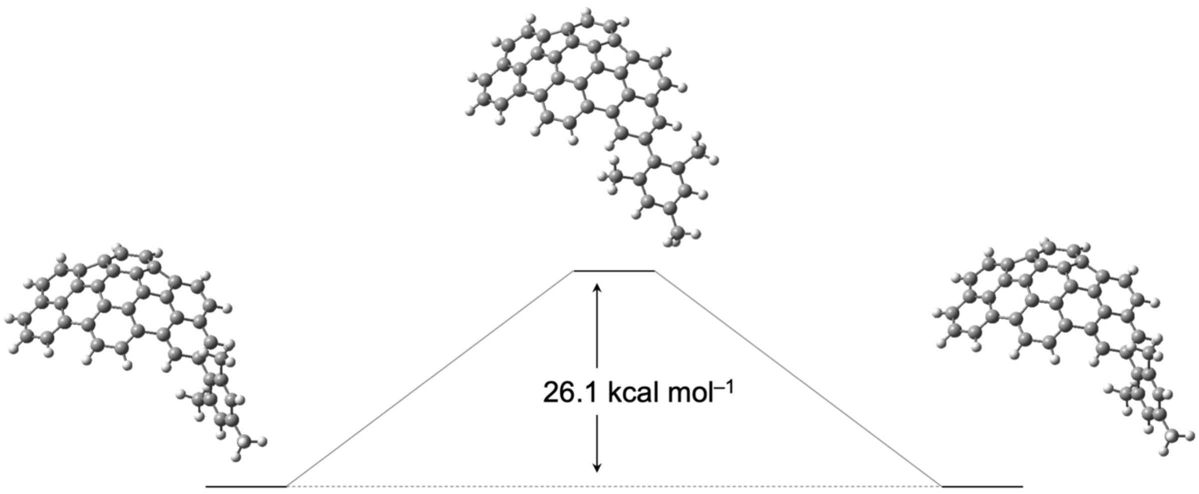

Supplementary Figure 25. Calculated rotation behavior of the mesityl group of **9** (CAM-B3LYP/6-311+G(d,p)//B3LYP/6-31G(d)).

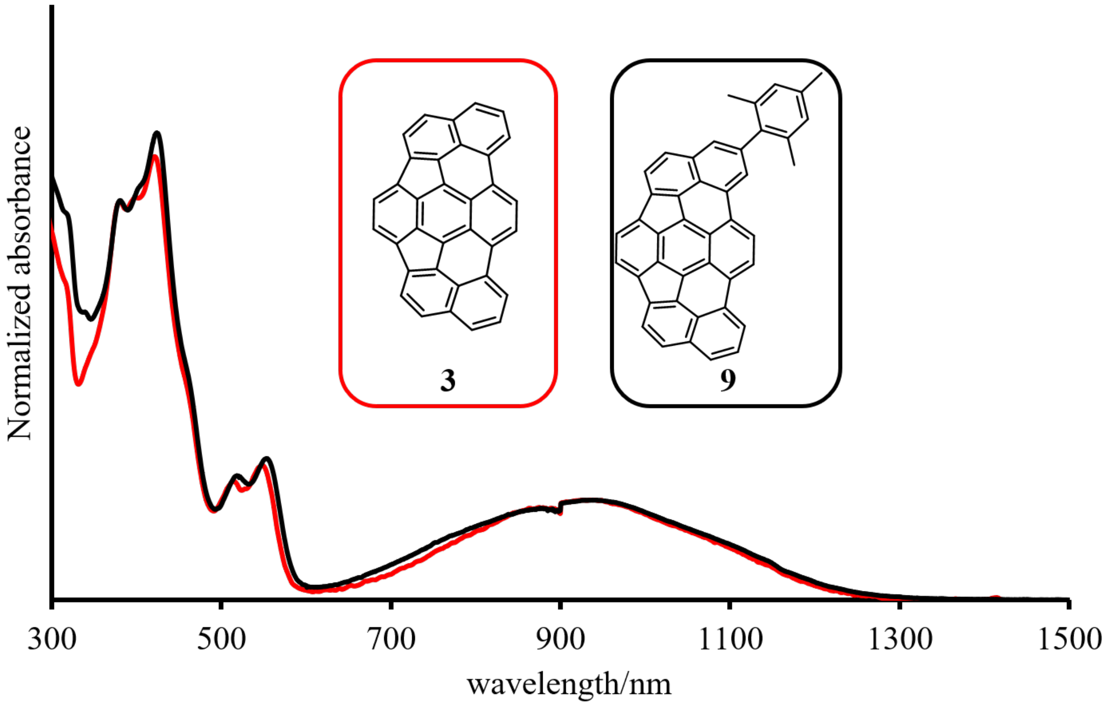

Supplementary Figure 26. UV/vis/NIR absorption spectra of **3** and **9** in CH_2Cl_2.

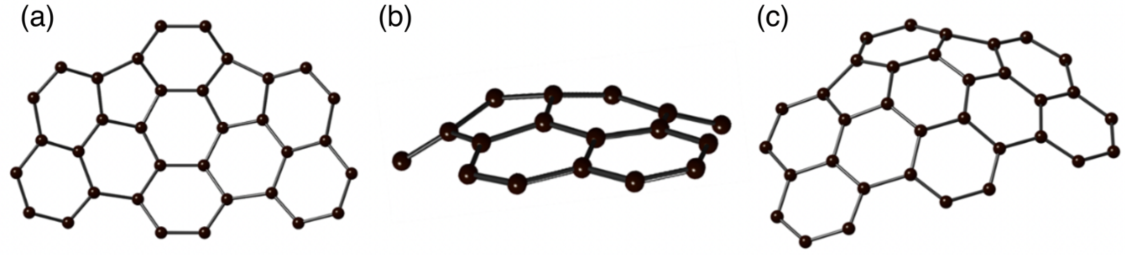

Supplementary Figure 27. Optimized structure of **3** at the CAM-B3LYP/6-311+G(d,p) level. (a) top view, (b) side view, and (c) allover view.

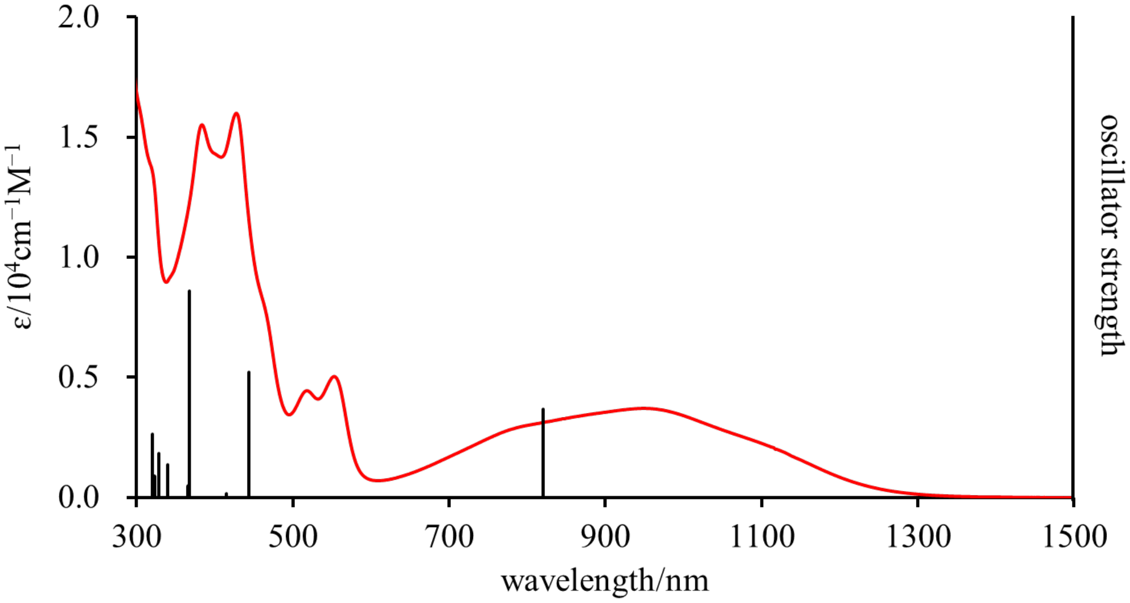

Supplementary Figure 28. Absorption spectrum of **3** in *o*-dichlorobenzene and the calculated oscillator strengths of **3**.

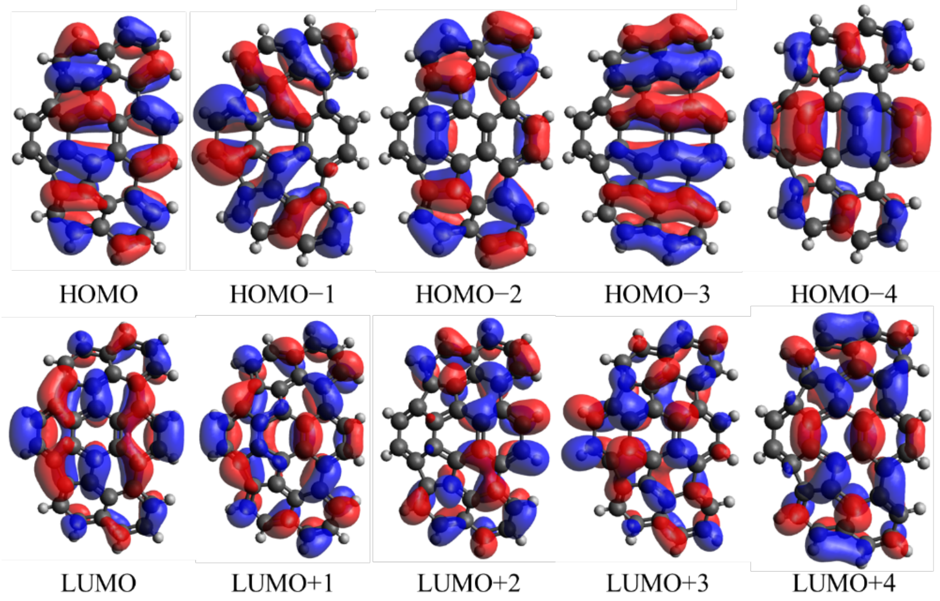

Supplementary Figure 29. Molecular orbitals of **3**.

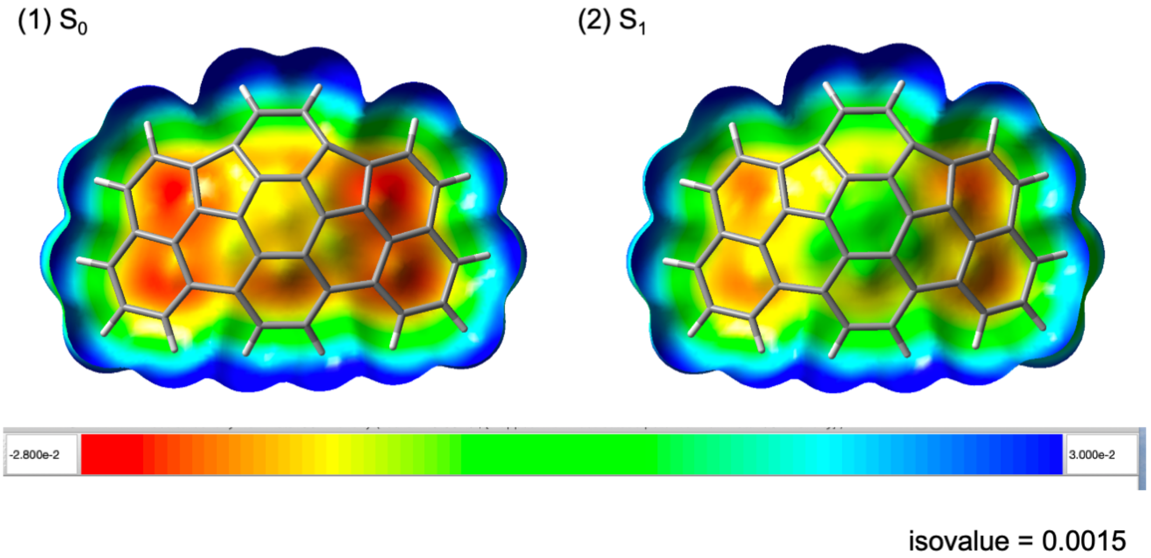

Supplementary Figure 30. Electrostatic potential maps of (1) S_0 state and (2) S_1 state of **3**.

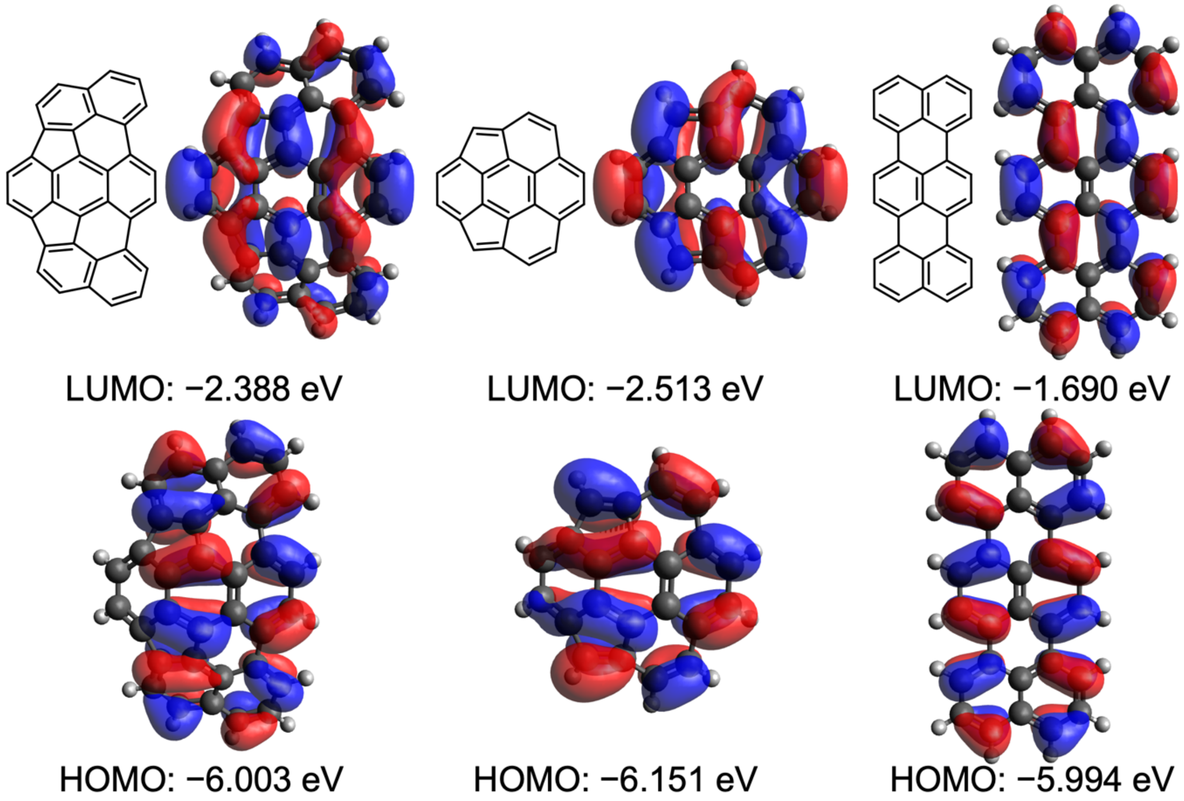

Supplementary Figure 31. Comparison of frontier orbitals of *as*-indacenoterrylene **3**, *as*-indacenopyrene and terrylene.

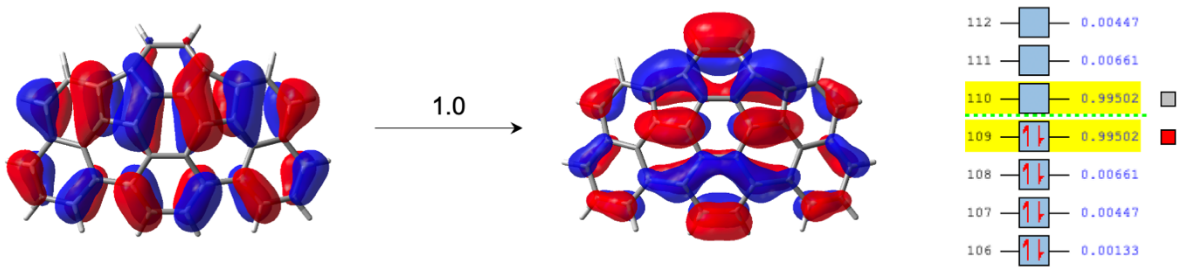

Supplementary Figure 32. Natural transition orbital analysis of *as*-indacenoterrylene **3** (CAM-B3LYP/6-311+G(d,p), isovalue = 0.03).

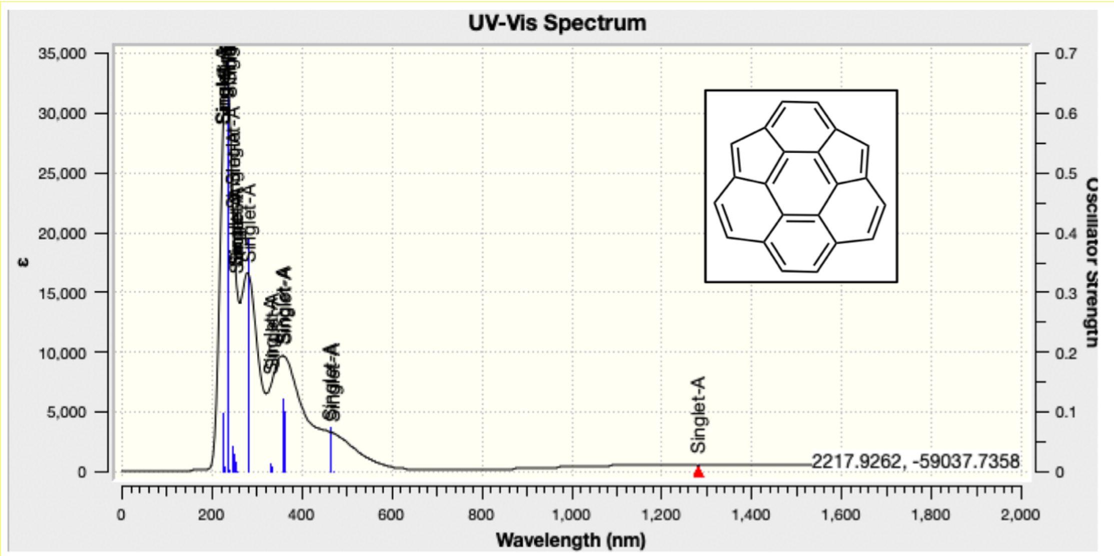

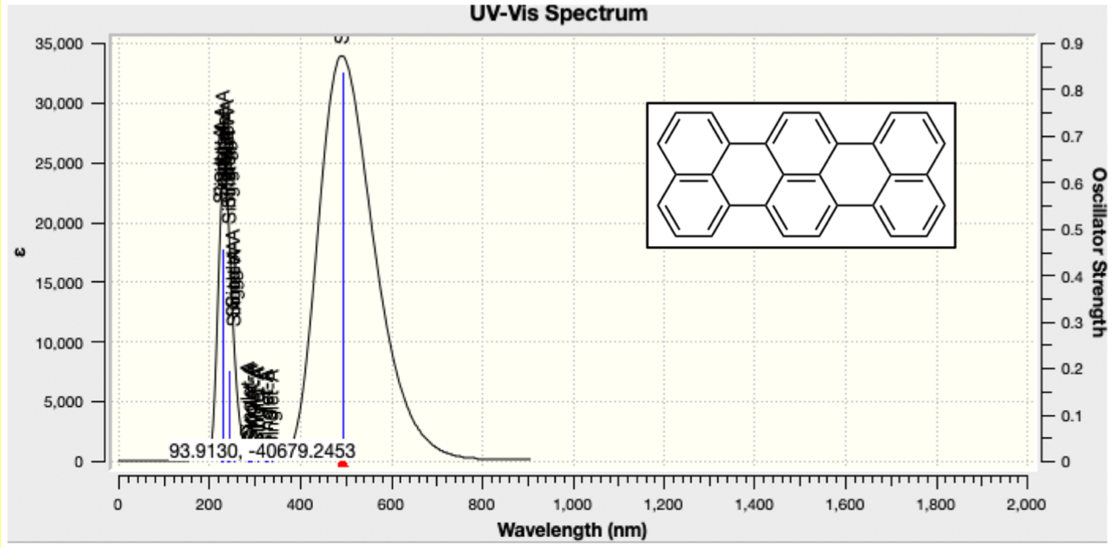

Supplementary Figure 33. Simulated absorption spectra of *as*-indacenopyrene and terrylene (CAM-B3LYP/6-311+G(d,p)).

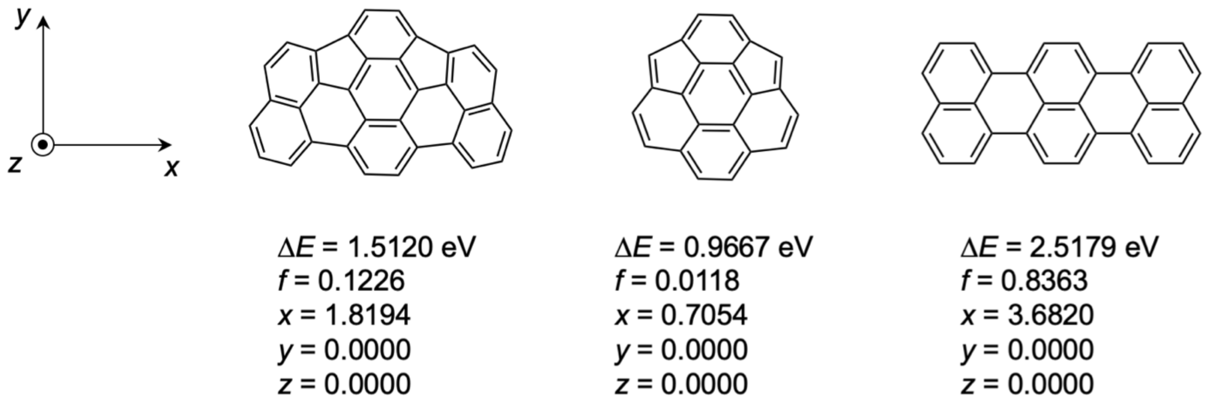

Supplementary Figure 34. Parameters for the S_0–S_1 transitions of *as*-indacenopyrene and terrylene (CAM-B3LYP/6-311+G(d,p)). Δ*E* = energy gap, *f* = oscillator strength, *x* = transition electric dipole moment along x-axis, *y* = transition electric dipole moment along y-axis and *z* = transition electric dipole moment along z-axis,

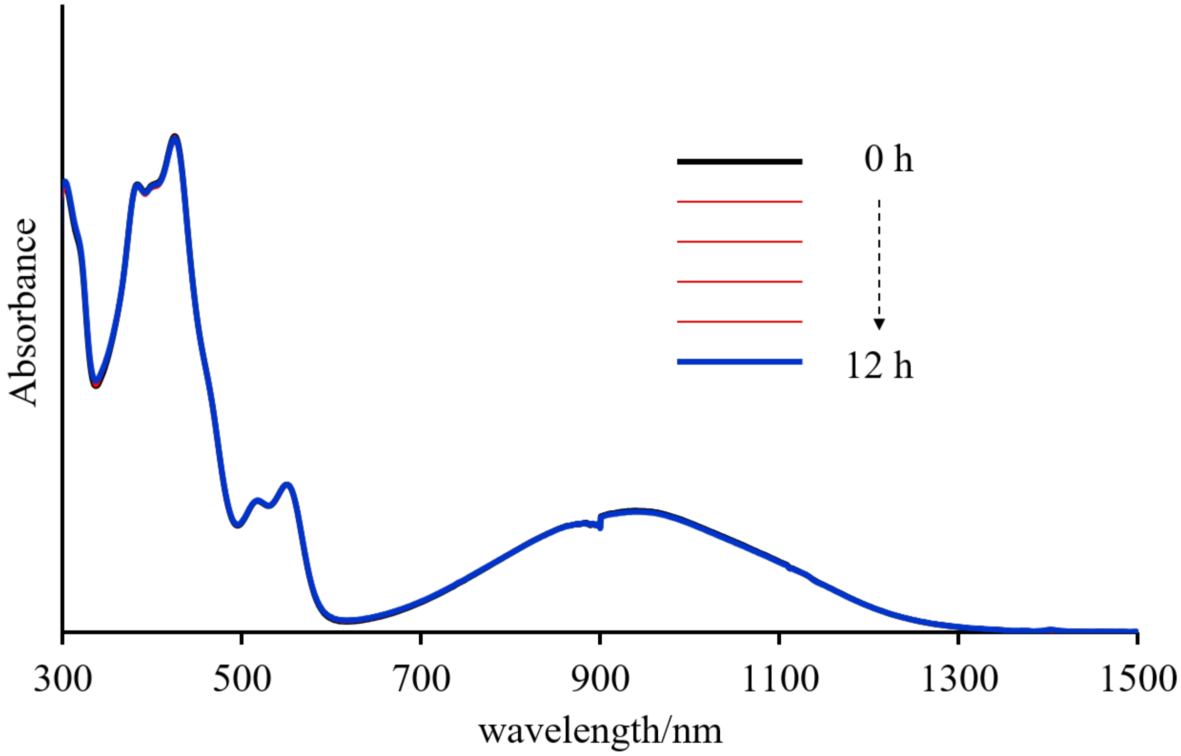

Supplementary Figure 35. UV/vis/NIR absorption spectra of **3** at 100 °C in *o*-dichlorobenzene.

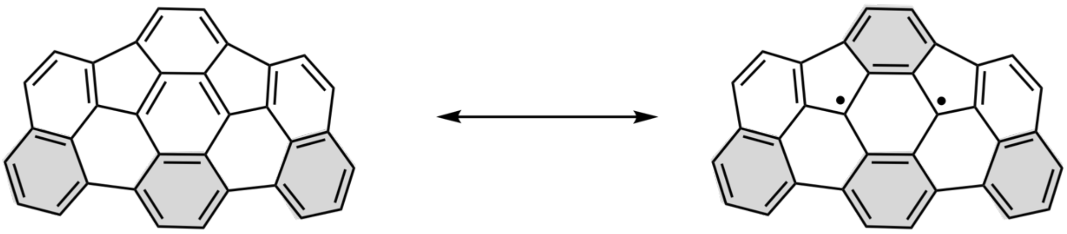

Supplementary Figure 36. Possible contributing structures in the resonance hybrid of **3**.

Supplementary Tables

Supplementary Table 1. Selected wavelengths, oscillator strengths and comparison of major electronic transitions of the **3**.

wavelength (nm)	oscillator strength	conditions
820.18	0.1226	108 -> 110 (-0.11071)<br>109 -> 110 (0.68873)
444.14	0.1740	108 -> 110 (0.68045)
414.78	0.0048	105 -> 110 (-0.26396)<br>107 -> 110 (0.62035)
367.30	0.2859	106 -> 110 (-0.38125)<br>109 -> 111 (0.54035)<br>109 -> 114 (-0.14315)
366.91	0.0149	105 -> 110 (0.42473)<br>107 -> 110 (0.20686)<br>109 -> 112 (0.36393)<br>109 -> 113 (0.31881)
340.14	0.0451	106 -> 110 (0.55947)<br>109 -> 111 (0.40362)
328.67	0.0605	104 -> 110 (0.22673)<br>105 -> 110 (-0.36195)<br>107 -> 110 (-0.21680)<br>107 -> 114 (-0.10657)<br>109 -> 112 (0.26519)<br>109 -> 113 (0.38309)
322.60	0.0285	103 -> 113 (-0.10114)<br>104 -> 110 (0.43287)<br>105 -> 110 (0.14864)<br>107 -> 111 (-0.13011)<br>108 -> 112 (-0.11936)<br>109 -> 112 (0.21907)<br>109 -> 113 (-0.35571)<br>109 -> 116 (0.22239)
321.43	0.0115	103 -> 110 (0.23003)<br>106 -> 110 (-0.11716)<br>107 -> 113 (-0.10176)<br>107 -> 116 (-0.10463)<br>109 -> 114 (0.60459)
318.13	0.0933	103 -> 110 (0.59511)<br>109 -> 114 (-0.24730)<br>109 -> 117 (0.10973)

Supplementary Table 2. NICS values of **3**.

	$NICS_{iso}$			$NICS_{zz}$		
	NICS(0)	$NICS(1)_{convex}$	$NICS(1)_{concave}$	NICS(0)	$NICS(1)_{convex}$	$NICS(1)_{concave}$
a	–3.94	–9.39	–8.80	6.85	–10.81	–7.98
b	–2.78	–6.61	–7.23	9.59	–6.49	–7.54
c	19.54	8.13	10.24	77.12	35.46	52.32
d	6.40	2.23	0.30	35.93	14.43	18.15
e	–0.66	–4.53	–6.82	13.54	0.83	2.22
f	–2.19	–1.89	–7.26	16.68	–1.13	–6.10
g	0.99	–1.89	–3.85	22.22	3.33	2.15

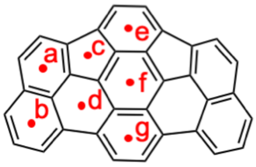

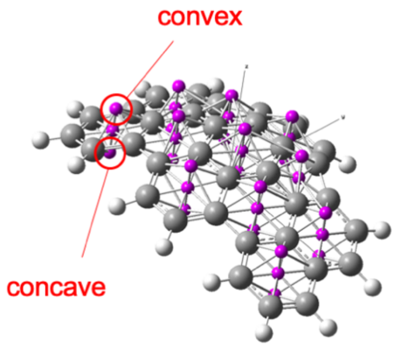

Supplementary Table 3. Cartesian coordinate and geometry of closed-shell singlet state of **3**.

C	-1.464657	2.67282	0.330119
C	-0.7286	3.689324	-0.235558
H	-1.208186	4.452977	-0.838184
C	0.728618	3.689284	-0.235628
H	1.208204	4.452909	-0.838295
C	1.46467	2.672793	0.33008
C	0.686373	1.676709	0.990952
C	1.340947	0.456629	1.002615
C	0.714031	-0.780179	0.947269
C	1.463064	-1.921191	0.51819
C	0.71218	-3.040315	0.220784
H	1.20089	-3.935995	-0.144348
C	-0.712261	-3.040307	0.22083
H	-1.201004	-3.935957	-0.144331
C	-1.463117	-1.921176	0.518286
C	-0.714056	-0.780172	0.947322
C	-1.340956	0.456646	1.002684
C	-0.686351	1.676707	0.990961
C	-2.629448	0.676509	0.442375
C	-2.805323	2.006142	0.071908
C	-4.020101	2.30745	-0.570781
H	-4.258551	3.317289	-0.884085
C	-4.929099	1.290643	-0.820265
H	-5.872394	1.537661	-1.294314
C	-4.659157	-0.081437	-0.542329
C	-5.499822	-1.171708	-0.881317
H	-6.471974	-0.988075	-1.324476
C	-5.069693	-2.455534	-0.664391
H	-5.719099	-3.283423	-0.923455
C	-3.779627	-2.745823	-0.158734
H	-3.476005	-3.783236	-0.083419
C	-2.915279	-1.736112	0.196052
C	-3.425649	-0.396596	0.06839
C	2.629434	0.676469	0.442296
C	2.805367	2.00612	0.071956
C	4.020238	2.307453	-0.570556
H	4.258715	3.317294	-0.88383
C	4.929186	1.290626	-0.820121
H	5.872503	1.537638	-1.294128
C	4.659187	-0.081478	-0.542294
C	5.499826	-1.171759	-0.881307
H	6.472024	-0.988139	-1.324371
C	5.069645	-2.455584	-0.66446
H	5.719037	-3.28348	-0.92353
C	3.779564	-2.745869	-0.158846
H	3.475945	-3.783281	-0.083497
C	2.915218	-1.736143	0.195911
C	3.425631	-0.396639	0.068319

Sum of electronic and thermal Free Energies = –1303.285549 Hartree

Supplementary Table 4. Cartesian coordinate and geometry of open-shell singlet state of **3**.

C	-1.464329	2.67376	0.330328
C	-0.728266	3.691761	-0.233967
H	-1.207972	4.456633	-0.834962
C	0.728196	3.69182	-0.233889
H	1.207891	4.456688	-0.834895
C	1.464273	2.673802	0.330351
C	0.686	1.678039	0.990461
C	1.341233	0.45697	1.003425
C	0.714154	-0.780224	0.948463
C	1.463079	-1.921142	0.520971
C	0.711536	-3.042647	0.227339
H	1.200482	-3.939707	-0.134076
C	-0.711427	-3.042658	0.227276
H	-1.20033	-3.939737	-0.134152
C	-1.463011	-1.921167	0.520854
C	-0.714132	-0.78024	0.948413
C	-1.341242	0.456936	1.003379
C	-0.686049	1.678028	0.990462
C	-2.62887	0.676451	0.443807
C	-2.80466	2.006733	0.072475
C	-4.018592	2.307436	-0.571422
H	-4.25684	3.317044	-0.885681
C	-4.927193	1.290096	-0.821756
H	-5.86987	1.536788	-1.297238
C	-4.657268	-0.082018	-0.543581
C	-5.496828	-1.172141	-0.884855
H	-6.468414	-0.988422	-1.329264
C	-5.066245	-2.456352	-0.668799
H	-5.714797	-3.284284	-0.929892
C	-3.777217	-2.746349	-0.161674
H	-3.473103	-3.783676	-0.087197
C	-2.913573	-1.736159	0.196107
C	-3.424448	-0.396931	0.068927
C	2.628865	0.676513	0.443864
C	2.804573	2.006769	0.072374
C	4.018419	2.307451	-0.571693
H	4.256625	3.31705	-0.886015
C	4.927091	1.290142	-0.821908
H	5.869746	1.536841	-1.297429
C	4.657257	-0.081948	-0.543555
C	5.49687	-1.172059	-0.884738
H	6.468426	-0.988328	-1.329205
C	5.066348	-2.456271	-0.668576
H	5.714929	-3.284194	-0.929623
C	3.777329	-2.746281	-0.161433
H	3.473239	-3.783613	-0.086931
C	2.913654	-1.736104	0.196303
C	3.424472	-0.396863	0.069029

Sum of electronic and thermal Free Energies = –1303.286913 Hartree

Supplementary Table 5. Cartesian coordinate and geometry of triplet state of **3**.

C	-1.454212	2.703045	0.330247
C	-0.712297	3.771241	-0.196057
H	-1.203491	4.575282	-0.732659
C	0.712298	3.771241	-0.196057
H	1.203493	4.575281	-0.732659
C	1.454213	2.703045	0.330248
C	0.677607	1.718717	0.970153
C	1.34839	0.465316	1.024972
C	0.717712	-0.790135	0.99224
C	1.455473	-1.915357	0.589586
C	0.686803	-3.105539	0.384435
H	1.193378	-4.030857	0.13853
C	-0.686804	-3.105539	0.384436
H	-1.193379	-4.030857	0.138531
C	-1.455474	-1.915357	0.589587
C	-0.717713	-0.790135	0.99224
C	-1.34839	0.465317	1.024972
C	-0.677606	1.718717	0.970153
C	-2.598315	0.671422	0.478303
C	-2.769593	2.030856	0.074383
C	-3.968249	2.308697	-0.592217
H	-4.20646	3.309931	-0.932451
C	-4.866001	1.276915	-0.856884
H	-5.794343	1.515006	-1.364282
C	-4.596275	-0.102446	-0.575973
C	-5.401713	-1.181968	-0.972834
H	-6.354994	-1.001213	-1.456481
C	-4.959808	-2.482944	-0.771877
H	-5.590265	-3.307399	-1.082928
C	-3.707767	-2.765208	-0.226561
H	-3.392493	-3.798768	-0.154192
C	-2.8574	-1.735994	0.200477
C	-3.37896	-0.410277	0.078349
C	2.598315	0.671421	0.478303
C	2.769594	2.030855	0.074385
C	3.968251	2.308696	-0.592214
H	4.206463	3.309931	-0.932447
C	4.866002	1.276914	-0.856882
H	5.794344	1.515005	-1.36428
C	4.596275	-0.102447	-0.575972
C	5.401713	-1.181969	-0.972835
H	6.354994	-1.001214	-1.456481
C	4.959806	-2.482946	-0.771879
H	5.590263	-3.3074	-1.082931
C	3.707765	-2.765209	-0.226563
H	3.392491	-3.798769	-0.154195
C	2.857399	-1.735994	0.200475
C	3.378959	-0.410278	0.078349

Sum of electronic and thermal Free Energies = –1303.276321 Hartree

Supplementary Methods

Synthesis of compounds. 1,4,5,8-Tetrachloro-9,10-*cis*-dimethoxy-9,10-dinaphthyl-9,10-dyhydroanthracene (**5**). A 300 mL three-necked round-bottom flask was evacuated and then refilled with N_2, To the flask, 1-bromonaphthalene (2.48 g, 12.0 mmol) and Et_2O (dry and degassed) were added. After stirring at 0 °C, *n*BuLi (1.55 M, 8.4 mL) was added. The reaction mixture was stirred at 0 °C, for 30 min. 1,4,5,8-Tetrachloroanthraquinone (**4**) (1.38 g, 3.99 mmol) was added to the mixture. The reaction mixture was stirred at 0 °C for 2 h and at room temperature for 5 h. The reaction was quenched with water (3.0 mL). Et_2O was removed by rotary evaporator. The solid materials were collected by filtration. They were washed with water and hexane. The solid materials were dried in *vacuo* at 60 °C. A 300 mL three-necked round-bottom flask was evacuated and then refilled with N_2. To the flask, the crude mixture and DMF (80 ml, dry and degassed) were added. The mixture was stirred at room temperature. To the mixture, MeI (2.84 g, 20 mmol) and NaH (60 %, dispersion in paraffin) (800 mg, 20 mmol) were added. The reaction mixture was stirred for 12 h at room temperature. The reaction was quenched by addition of water (80 mL). The precipitates were collected by filtration. The solid materials were washed with water and hexane. The solid materials were dried in *vacuo* at 60 °C. They included **5** and **6**. Compound **5** showed higher solubility than **6** in CH_2Cl_2. Successive purification by reprecipitation in CH_2Cl_2 afforded **5** in 22% yield (559 mg, 0.887 mmol) as a white solid. ^{1}H NMR ($CDCl_3$): δ 9.76 (d, J = 8.0 Hz, 2H), 7.79 (dd, J = 7.5 Hz, 2H), 7.72 (d, J = 8.0 Hz, 2H), 7.58 (d, J = 7.5 Hz, 2H), 7.52 (dt, J = 7.5 Hz, 2H), 7.44 (dt, J = 7.5 Hz, 2H), 7.22 (t, J = 8.0 Hz, 2H), 7.18 (s, 4H), 3.18 (s, 6H) ppm; ^{13}C NMR ($CDCl_3$): δ 139.6, 139.1, 137.4, 134.5, 133.5, 133.2, 132.0, 129.5, 129.4, 128.7, 124.8, 124.7, 123.2, 87.0, 51.5 ppm; HRMS (APCI): $[M+H]^+$ Calcd for $C_{36}H_{24}{}^{35}Cl_4O_2$ 629.0603; Found 629.0587.

Dimethoxy-substituted dihydro-*as*-indaceno[3,2,1,8,7,6-*ghijklm*]terrylene (**7**). The synthetic procedure is shown in the main text. ^{1}H NMR ($CDCl_3$) δ 7.81 (d, J = 7.5 Hz, 2H), 7.69 (s, 2H), 7.66 (d, J = 8.5 Hz, 2H), 7.57 (d, J = 7.5 Hz, 2H), 7.51 (d, J = 8.5 Hz, 2H), 7.28 (dd, J =7.5 Hz, 2H), 7.21 (s, 2H), 3.66 (s, 6H) ppm; ^{13}C NMR ($CDCl_3$): δ 150.3, 140.7, 140.2, 134.2, 134.1, 132.1, 131.7, 129.8, 128.0, 127.9 (overlap), 127.1, 126.2, 124.9, 122.1, 119.7, 76.4, 53.6 ppm; HRMS (APCI): $[M+H]^+$ Calcd for $C_{36}H_{20}O_2$ 485.1536; Found 485.1543.

Dihydro-*as*-indaceno[3,2,1,8,7,6-*ghijklm*]terrylene (**8**). The synthetic procedure is shown in the main text. ^{1}H NMR ($CDCl_3/CS_2$) δ 7.68 (d, J = 7.3 Hz, 2H), 7.53–7.51 (m, 6H), 7.48 (s, 2H), 7.23 (dd, J = 7.3 Hz, 2H), 5.91 (s, 2H) ppm; ^{13}C NMR ($CDCl_3/CS_2$): δ 148.5, 145.8, 139.7, 133.1, 133.0, 132.8, 131.3, 128.5, 127.3, 126.8, 126.5, 124.7, 123.7, 123.7, 120.2, 120.2, 45.9 ppm; HRMS (APCI): $[M+H]^+$ Calcd for $C_{34}H_{16}$ 425.1325; Found 425.1345.

as-Indaceno[3,2,1,8,7,6-*ghijklm*]terrylene (**3**). The synthetic procedure is shown in the main text. ^{1}H NMR ($CDCl_3/CS_2$) δ 7.21 (d, J = 7.5 Hz, 2H), 6.99 (d, J = 8.0 Hz, 2H), 6.90 (s, 2H), 6.88 (t, J = 7.5 Hz, 2H), 6.87 (d, J = 8.0 Hz, 2H), 6.80 (d, J = 8.0 Hz, 2H), 6.78 (s, 2H) ppm; HRMS (APCI): $[M+H]^+$ Calcd for $C_{34}H_{14}$ 423.1168; Found 423.1184 (The ^{13}C NMR spectrum was not obtained due to the low solubility.).

3-(4,4,5,5-tetramethyl-1,3,2-dioxaboronate)-dimethoxy-*as*-indacenodihydroterrylene (**10**). A Schlenk tube containing B_2pin_2 (100 mg, 0.394 mmol), $[Ir(cod)OMe]_2$ (26.6 mg, 40.1 μmol) and 4,4'-di-*tert*-butyl-2,2'-bipyridyl (20.4 mg, 76.0 μmol) was evacuated and then refilled with N_2. To the tube, THF (8.0 mL, dried and degassed) was added. The reaction mixture was stirred at room temperature until the color was changed to dark red. To the mixture, **7** (187 mg, 0.396 mmol) was added. The reaction mixture was stirred at 80 °C for 2 h. The mixture was poured into CH_2Cl_2. The resulting mixture was passed through short silica-gel (CH_2Cl_2 as an eluent). The solvent was removed by rotary evaporator. Purification by size exclusion chromatography ($CHCl_3$ as eluent) afforded the mono-borylated compound **10** in 25% yield (60.3 mg, 98.8 μmol) as a yellow solid. ^{1}H NMR ($CDCl_3$) δ 8.17 (s, 1H), 8.11 (s, 1H), 7.81 (dd, J = 7.0 Hz, 2H), 7.69 (d, J = 8.0 Hz, 2H), 7.66 (d, J = 8.0 Hz, 1H), 7.57 (d, J = 8.0 Hz, 1H), 7.51 (d, J = 1.5 Hz, 1H), 7.50 (d, J = 1.5 Hz, 1H), 7.29 (dd, J = 7.5 Hz, 1H), 7.22 (d, J = 1.5 Hz, 2H), 3.64 (s, 3H), 3.61 (s, 3H), 1.39 (s, 6H), 1.39 (s, 6H) ppm; ^{13}C NMR ($CDCl_3$): δ 150.6, 150.3, 141.3, 140.7, 140.4, 140.3, 140.2, 139.9, 136.3, 134.3, 134.1, 134.0, 132.1, 131.8, 131.4, 130.9, 130.5, 129.8, 129.2, 128.0, 127.9, 127.9, 127.1, 126.4, 126.1, 126.0, 125.0, 124.8, 122.0, 119.7, 119.7, 119.7, 84.2, 53.6, 53.5, 25.2, 25.0 ppm; HRMS (APCI): $[M+H]^+$ Calcd for $C_{42}H_{31}{}^{11}BO_4$ 611.2395; Found 611.2411.

3-(2,4,6-Trimethylphenyl)-dimethoxy-*as*-indacenodihydroterrylene (**11**). A Schlenk tube containing **10** (15.0 mg, 24.6 μmol), 2-bromomesitylene (7.77 mg, 39.0 μmol), $Pd_2(dba)_3$•$CHCl_3$ (2.60 mg, 2.51 μmol), SPhos (1.92 mg, 4.68 μmol) and K_2CO_3 (34.4 mg, 0.249

mmol) was evacuated and the refilled with N_2. To the tube, toluene (3.0 mL, degassed) and H_2O (0.30 mL, degassed) were added. The reaction mixture was stirred for 5 h at 90 °C and passed through Celite and dried over Na_2SO_4. After removing the solvent, Purification by silica-gel column chromatography (CH_2Cl_2/hexane as eluent) afforded the **11** in 36% yield (5.36 mg, 8.90 μmol) as a yellow solid. ^{1}H NMR ($CDCl_3$) δ 7.80 (d, J = 7.0 Hz, 1H), 7.69 (d, J = 8.5 Hz, 1H), 7.66 (d, J = 1.5 Hz, 1H), 7.65 (d, J = 1.5 Hz, 1H), 7.65 (d, J = 8.5 Hz, 1H), 7.62 (s, 1H), 7.57 (d, J = 8.5 Hz, 1H), 7.53 (d, J = 8.5 Hz, 1H), 7.51 (d, J = 7.51 Hz, 1H), 7.34 (s, 1H), 7.27 (d, J = 7.0 Hz, 1H), 7.00 (s, 1H), 6.96 (s, 1H), 3.73 (s, 3H), 3.68 (s, 3H), 2.36 (s, 3H), 2.15 (s, 3H), 1.93 (s, 3H) ppm; ^{13}C NMR ($CDCl_3$): δ 150.3, 150.2, 140.8, 140.2, 140.1, 139.9, 138.8, 138.0, 137.1, 136.6, 136.0, 134.5, 134.3, 134.2, 133.9, 132.3, 132.0, 131.9, 131.6, 129.8, 129.8, 128.4, 128.0, 128.0, 127.8, 127.0, 126.9, 126.3, 126.2, 124.9, 124.8, 124.5, 122.1, 119.9, 119.7, 76.4, 76.4, 21.2, 21.1, 20.8 ppm; HRMS (APCI): $[M+H]^+$ Calcd for $C_{45}H_{30}O_2$ 603.2319; Found 603.2296.

3-(3,5-Di-*tert*-butylphenyl)-dimethoxy-*as*-indacenodihydroterrylene (**12**). A Schlenk tube containing **10** (10.4 mg), 1-bromo-3,5-di-*tert*-butylbenzene (9.01 mg, 33.4 μmol), $Pd(PPh_3)_4$ (2.62 mg, 2.25 μmol), SPhos (2.43 mg, 5.90 μmol) and K_2CO_3 (22.7 mg, 0.166 mmol) was evacuated and the refilled with N_2. To the tube, toluene (2.0 mL, degassed) and H_2O (0.2 mL, degassed) were added. The reaction mixture was stirred for overnight at 90 °C and passed through Celite and dried over Na_2SO_4. After removing the solvent, Purification by silica-gel column chromatography (CH_2Cl_2/hexane as eluent) afforded the **12** in 27% yield (3.02 mg, 4.46 μmol) as a yellow solid. ^{1}H NMR ($CDCl_3$) δ 8.01 (s, 1H), 7.82 (d, J = 7.0 Hz, 1H), 7.76 (s, 1H), 7.73 (t, J = 8.0 Hz, 1H), 7.73 (d, J = 8.0 Hz, 1H), 7.69 (d, J = 8.5 Hz, 1H), 7.67 (d, J = 8.5 Hz, 1H), 7.58 (d, J = 8.0 Hz, 1H), 7.53 (d, J = 7.0 Hz, 2H), 7.52 (s, 1H), 7.50 (d, J = 10.5 Hz, 2H), 7.50 (d, J = 10.5 Hz, 1H), 7.29 (d, J = 7.5 Hz, 1H), 7.23 (s, 2H), 3.70 (s, 3H), 3.66 (s, 3H), 1.41 (s, 18H) ppm; ^{13}C NMR ($CDCl_3$): δ 151.5, 150.4, 150.3, 141..7, 140.9, 140.8, 140.6, 140.3, 140.2, 140.1, 139.9, 134.6, 134.3, 134.1, 134.0, 132.2, 132.1, 132.0, 131.7, 129.9, 129.8, 128.0, 127.9, 127.2, 127.1, 126.3, 126.2, 126.0, 124.9, 124.8, 123.1, 122.2, 122.0, 121.8, 120.0, 119.7 76.4, 53.7, 53.6, 35.2, 31.7 ppm; HRMS (APCI): $[M+H]^+$ Calcd for $C_{50}H_{40}O_2$ 673.3101; Found 673.3070.

3-(2,4,6-Trimethylphenyl)-*as*-indacenodihydroterrylene (**13**). A 50 mL two necked round-bottom flask containing **11** (21.7 mg, 36.1 μmol) was evacuated and then refilled with N_2. To the tube, CH_2Cl_2 (30.0 mL, dried and degassed) and $HSiEt_3$ (210 mg, 1.81 mmol) were added. After stirring, TfOH (27.2 mg, 0.181 mmol) was added. The mixture was stirred at room temperature for 1 h. The reaction was quenched with NEt_3. The mixture was passed through short silica-gel (CH_2Cl_2 as an eluent). The solvent was removed by rotary evaporator. Purification by washing the residue with MeOH and cold hexane afforded the **7** in 98% yield (19.1 mg, 35.2 μmol) as a yellow solid. ^{1}H NMR ($CDCl_3$) δ 7.69 (d, J = 6.5 Hz, 1H), 7.58 (d, J = 7.5 Hz, 1H), 7.55 (s, 2H), 7.54 (d, J = 6.5 Hz, 1H), 7.52 (d, J = 10.0 Hz, 2H), 7.50 (d, J = 10.0 Hz, 2H), 7.46 (d, J = 7.5 Hz, 1H), 7.39 (s, 2H), 7.32 (s, 1H), 7.23 (t, J = 8.0 Hz, 1H), 7.00 (s, 1H), 6.96 (s, 1H), 5,96 (d, J = 2.5 Hz, 1H), 5.92 (d, J = 2.5 Hz, 1H), 2.36 (s, 3H), 2.16 (s, 3H), 1.94 (s, 3H) ppm; ^{13}C NMR ($CDCl_3$): δ 148.7, 148.7, 146.1, 146.0, 139.9, 139.9, 139.8, 139.8, 139.4, 139.1, 137.0, 136.5, 136.0, 133.5, 133.4, 133.4, 133.2, 133.1, 132.9, 131.6, 131.4, 128.6, 128.4, 128.3, 127.7, 127.5, 127.4, 127.0, 126.9, 126.6, 125.0, 124.8, 123.8, 123.8, 122.7, 120.5, 120.4, 120.3, 46.0, 21.2, 20.9, 20.8 ppm; HRMS (APCI): $[M+H]^+$ Calcd for $C_{43}H_{26}$ 543.2107; Found 543.2131.

3-(2,4,6-Trimethylphenyl)-*as*-indacenoterrylene (**9**). A 50 mL two necked round-bottom flask containing **13** (13.7 mg, 25.2 μmol) was evacuated and then refilled with N_2. To the tube, CH_2Cl_2 (30.0 mL, dried and degassed) was added. After stirring, a solution of DDQ in CH_2Cl_2 (2.36 mM, 12.5 mL) was added. The mixture was stirred at room temperature for 1 h. The mixture was passed through short silica-gel (CH_2Cl_2 as an eluent). The solvent was removed by rotary evaporator. Purification by washing the residue with MeOH and cold hexane afforded the **9** in 67% yield (9.20 mg, 17.0 μmol) as a dark red solid. ^{1}H NMR (500 MHz, $CDCl_3$) δ 7.20 (d, J = 7.5 Hz, 1H), 7.03 (s, 1H), 7.01 (d, J = 8.5 Hz, 1H), 6.94–6.79 (m, 10H), 6.76 (s, 2H), 2.33 (s, 3H), 2.10 (s, 3H), 2.07 (s, 3H) ppm; ^{13}C NMR (126 MHz, $CDCl_3$): δ = 143.9, 143.7, 143.1, 142.1, 142.0, 140.7, 138.9, 137.6, 137.0, 136.2, 135.9, 135.1, 135.0, 135.0, 134.6, 134.5, 134.2, 134.0, 133.5, 133.2, 130.1, 129.0, 128.5, 128.5, 128.3 (overlap), 127.8, 127.4, 127.3, 126.8, 126.0, 125.8, 125.5, 123.5, 122.4, 122.2, 121.6, 77.4, 21.2, 20.9, 20.7 ppm; HRMS (APCI): $[M+H]^+$ Calcd for $C_{43}H_{24}$ 541.1951; Found 541.1963.

Crystal Data. 1,4,5,8-Tetrachloro-9,10-*cis*-dimethoxy-9,10-dinaphthyl-9,10-dyhydroanthracene (**5**). Single crystals were obtained by vapor diffusion of hexane into a dichloromethane solution of **5**. $C_{36}H_{24}Cl_4O_2$, M_w = 698.76, monoclinic, space group $P2_1/c$, a = 12.9739(3), b = 13.4661(3), c = 17.9708(3) Å, β = 97.804(2), V = 3870.1(5) Å^3, Z = 8, D_{calc} = 1.457 g/cm^3, T = 93(2) K, R = 0.0662 ($I > 2.0\ \sigma(I)$), wR = 0.1732 (all data), GOF = 1.049. CCDC number: 2004717.

Dimethoxy-substituted dihydro-*as*-indaceno[3,2,1,8,7,6-*ghijklm*]terrylene (**7**). Single crystals were obtained by vapor diffusion of hexane into a dichloromethane solution of **7**. $C_{36}H_{20}O_2$, M_w = 484.52, orthorhombic, space group $Pca2_1$, a = 11.2176(3), b = 22.4515(6), c = 9.1081(3) Å, V = 2293.89(12) Å^3, Z = 4, D_{calc} = 1.403 g/cm^3, T = 106(2) K, R = 0.0512 ($I > 2.0\ \sigma(I)$), wR = 0.1107 (all data), GOF = 1.106. CCDC number: 2004720.

Dihydro-*as*-indaceno[3,2,1,8,7,6-*ghijklm*]terrylene (**8**). Single crystals were obtained by liquid-liquid diffusion method of hexane into its o-dichlorobenzene solution of **8**. $C_{34}H_{16}$, M_w = 424.47, orthorhombic, space group $Fdd2$, a = 22.5417(18), b = 41.554(3), c = 4.1316(3) Å, V = 3870.1(5) Å^3, Z = 8, D_{calc} = 1.457 g/cm^3, T = 93(2) K, R = 0.0662 ($I > 2.0\ \sigma(I)$), wR = 0.1732 (all data), GOF = 1.049. CCDC number: 2004719.

3-(3,5-Di-*tert*-butylphenyl)-dimethoxy-*as*-indacenodihydroterrylene (**12**). Single crystals were obtained by liquid-liquid diffusion method of MeOH into its dichloromethane solution of **12**. $C_{50}H_{40}O_2$, M_w = 754.74, monoclinic, space group Pn, a = 10.2966(3), b = 14.4638(5), c = 12.8787(3) Å, β = 102.676(3), V = 1871.25(10) Å^3, Z = 2, D_{calc} = 1.345 g/cm^3, T = 93(2) K, R = 0.0574 ($I > 2.0\ \sigma(I)$), wR = 0.1537 (all data), GOF = 1.054. CCDC number: 2004718.
